# Dynamic Fusion of Genomics and Functional Network Connectivity in UK Biobank Reveals Schizophrenia‐Related SNP Manifolds

**DOI:** 10.1002/hbm.70530

**Published:** 2026-04-20

**Authors:** Jiayu Chen, Armin Iraji, Zening Fu, Marlena Duda, Pablo Andrés‐Camazón, Bishal Thapaliya, Jingyu Liu, Vince D. Calhoun

**Affiliations:** ^1^ Tri‐Institutional Center for Translational Research in Neuroimaging and Data Science (TReNDS): (Georgia State University, Georgia Institute of Technology, and Emory University) Atlanta Georgia USA; ^2^ Department of Computer Science Georgia State University Atlanta Georgia USA; ^3^ Department of Child and Adolescent Psychiatry Institute of Psychiatry and Mental Health, Hospital General Universitario Gregorio Marañón, IiSGM Madrid Spain

**Keywords:** dynamic, functional network connectivity, fusion, genomics, independent components analysis (ICA), schizophrenia

## Abstract

Many mental disorders show strong genetic influence. In parallel, dynamic functional network connectivity (dFNC) has shown high sensitivity to brain changes related to mental disorders. However, previous studies linking dFNC to genetics largely follow a paradigm to identify associations between one set of genetic factors and multiple sets of connectivity features from different dFNC states, ignoring the potential variability in genetic correlates across states. We propose a novel joint ICA (jICA)‐based “dynamic fusion” framework to identify dynamically tuned genetic manifolds. A sliding window approach was utilized to estimate four dFNC states and compute subject‐level state‐average dFNC (sa‐dFNC) features. The sa‐dFNC features of each state were combined with schizophrenia risk single nucleotide polymorphisms (SNPs) within a jICA fusion framework, resulting in four parallel fusions in 32,861 individuals of the UK Biobank cohort. The extracted four sets of joint SNP‐dFNC components were further validated for clinical relevance in a combined schizophrenia cohort of 820 individuals (348 patients). The similarity of SNP‐dFNC components across four parallel fusions was evaluated as a measure of state variability. We observed a mixture of “state‐invariant” and “state‐variant” components for SNP and dFNC modalities. Particularly, the schizophrenia‐related state‐variant SNP components, or manifolds, complemented each other by capturing different SNPs involved in the same biological functions, revealing a partition of genomic risk particularly elicited by the dynamics of brain function. By augmenting the SNP factors to state‐variant manifolds, this dynamic fusion framework promises additional insights into the underlying genetic risk of disease‐related alterations in dynamic brain function.

## Graphical abstract

A joint ICA (jICA)‐based “dynamic fusion” framework to identify dynamically tuned SNP manifolds by linking static SNPs to dynamic functional information of the brain.

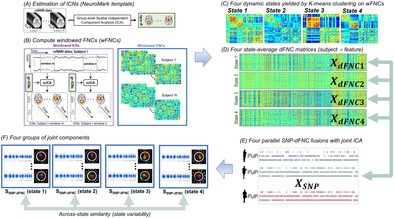

## 1 Introduction

It has been well established that many psychiatric and neurological disorders show significant heritability (Sullivan et al. 2003; Glahn et al. 2007; Anttila et al. 2018; Pettersson et al. 2019). Family and twin studies have suggested heritability rates ranging from 0.80 for schizophrenia (SZ) (Sullivan et al. 2003), to 0.90 for autism (ASD) (Sandin et al. 2017), and 0.60–0.80 for Alzheimer's disease (AD) (Gatz et al. 2006). More recently, large‐scale genome‐wide association studies (GWASs) have confirmed the polygenic nature of brain disorders, and significant estimates have been consistently reported across studies for common‐variant‐based heritability, varying from 0.10 for AD to 0.28 for obsessive‐compulsive disorder (OCD), and consistently 0.23–0.24 for SZ (Lee et al. 2013; Pettersson et al. 2019). Understanding how genetic factors influence predisposition, disease progression, and response to treatment is crucial in advancing personalized healthcare and precision medicine (Ashley 2016; Freudenberg‐Hua et al. 2018; Gandal et al. 2016).

A common notion is that in brain disorders, genetic factors exert influence on clinical manifestations by affecting the brain. The past decade has seen great efforts to unravel brain disorders by examining the relationship between genetic variants and changes in the brain, known as imaging genomics (Shen and Thompson 2020), so that brain disorders may be described from a multiscale multidimensional perspective for a comprehensive understanding of its underlying biology (Insel et al. 2010; Cuthbert 2014; Cuthbert and Insel 2013). The hope is that such a dimensional framework would allow detecting and quantifying risk at an early stage and stratifying individuals based on biological underpinnings, ultimately leading to more efficient individualized treatment (Insel 2014; Insel and Cuthbert 2015).

Dynamic functional network connectivity (dFNC) measures time‐resolved functional coupling between intrinsic brain connectivity networks. Mapping intrinsic connectivity networks (ICNs) has been shown to provide a potentially mechanistic framework for understanding human behavior and mental disorders (Mo et al. 2024; Zhang et al. 2024; Cheng et al. 2025; Li et al. 2025). Compared to conventional functional connectivity analysis, dFNC provides rich information that describes how functional interactions vary across time and has proven to identify disease‐related changes in the brain (Allen et al. 2014; Calhoun et al. 2014; Damaraju et al. 2014). One well‐established strategy to characterize dFNC is the sliding window approach, which captures the flow of functional connectivity through a sequence of windowed FNCs (wFNCs) (Iraji et al. 2021), and allows for estimating stable representative connectivity patterns, known as dFNC states (Abrol et al. 2017; Miller et al. 2018), for further investigation on clinical relevance (Allen et al. 2014; Miller et al. 2016). The dFNC approach has proven to yield highly informative neurobiological markers for disease discrimination, for example, classifying SZ, bipolar disorder, autism, and mild traumatic brain injury (Rashid et al. 2016; Rabany et al. 2019; Vergara et al. 2018), and significantly outperform static FNC in classifying patients with SZ and bipolar disorder (Rashid et al. 2016).

While dFNC effectively captures disease‐related alterations, its genetic correlates are understudied. The GWASs of brain imaging phenotypes conducted on the UK Biobank (UKB) data indicate that despite not showing as high heritability as structural phenotypes, 235 out of 1771 tested functional connectivity pairs showed significant heritability (Elliott et al. 2018). In addition, when independent component analysis (ICA) was applied to the 1771 connectivity pairs, the resulting connectivity components showed much stronger heritability (Elliott et al. 2018). These observations motivate further elucidations on genetic profiles underlying functional connectivity, particularly those disrupted in SZ. Prior work linking genetics to dFNC typically follows a paradigm to identify associations between one set of single nucleotide polymorphism (SNP) factors and multiple sets of connectivity features from different dynamic states. While this is a powerful approach, it ignores the possibility that there might be locally optimizable SNP‐dFNC factors, such that unique SNP factors might be discovered by separately linking SNPs to each dFNC state.

The current work aims to address the gap by proposing a novel joint ICA (jICA)‐based “dynamic fusion” framework to identify manifolds of SNP‐dFNC association through parallel data fusions between SZ‐risk SNPs and individual dFNC states. We leveraged the most recent psychiatric genomic consortium (PGC) GWAS of SZ to select out SNPs conferring risk for SZ (Trubetskoy et al. 2022) and jointly analyzed them with dFNC features estimated using the NeuroMark pipeline (Du et al. 2020) in the UKB cohort. The identified joint SNP‐dFNC components were further investigated for SZ relevance in a combined SZ cohort. We hypothesized that the joint SNP‐dFNC components would show different levels of state variability. To the best of our knowledge, this is the first work exploring the dynamically tuned SNP decompositions across multiple data fusions with dFNC. We hope that linking SNPs to dynamic functional brain information augments the SNP modality to across‐state manifolds, and thus provides a unique lens to elicit unique SNP correlates missed in previous work.

## 2 Methods and Materials

### 2.1 Participants

#### 2.1.1 UK Biobank

The current work leveraged the population‐based UKB cohort which recruited more than 500 K individuals across the United Kingdom (Bycroft et al. 2018; Littlejohns et al. 2020). The UKB study was approved by the North West Haydock Research Ethics Committee, and the data used in our work were obtained under application 34,175. Specifically, we used the imputed SNP data and resting‐state functional magnetic resonance images (rsfMRI) of 32,681 European ancestry individuals with both imaging and genetic modalities available after quality control (QC), including 15,357 males and 17,504 females, aged between 45 and 81 with a median of 64.

#### 2.1.2 SZ Cohorts

Additional SZ cohorts were utilized to validate the UKB findings for SZ relevance. For this purpose, three SZ cohorts were combined to boost power, including COBRE (Aine et al. 2017), FBIRN (Damaraju et al. 2014), and MPRC (Kochunov et al. 2017), resulting in 820 individuals with rsfMRI data after QC, including 348 individuals with SZ and 472 controls (62.80% males, aged between 16 and 79 with a median of 39). The institutional review board at each site approved the study, and all participants provided written informed consent. Details regarding recruitment and data collection can be found in previous publications (Damaraju et al. 2014; Kochunov et al. 2017; Yu et al. 2015; Chen et al. 2018; Skatun et al. 2018).

### 2.2 SNP Preprocessing and Prescreening

#### 2.2.1 SNP Data

The imputed SNP data released by UKB consisted of 487,320 individuals and ~96 million variants (v3_s487320). Details of genotyping and imputation can be found in the paper that describes the UKB genomic data (Bycroft et al. 2018).

#### 2.2.2 Preprocessing of SNP

In this study, we first identified the participants that passed the UKB quality control (sex mismatch, missing rate, and heterozygosity) and also had rsfMRI data available. We excluded SNPs with minor allele frequencies < 0.01 (Chen et al. 2019). Individual relatedness (identify‐by‐descent) was estimated using PLINK (Purcell et al. 2007). For each group of individuals who were second‐degree relatives or closer, only one individual was randomly selected and retained for subsequent analysis. Finally, we identified individuals of European ancestry to be those close (< 3SD) to the center of the “white” cluster as defined by the top four principal components (Chen et al. 2019, 2022).

#### 2.2.3 Prescreening

We selected SZ risk SNPs based on the 287 loci identified by the PGC GWAS of SZ (Trubetskoy et al. 2022), which were further pruned at *r* ^*2*^ < 0.2, a commonly used threshold for European populations (Galinsky et al. 2016), resulting in a total of 12,946 SNPs included in the dynamic fusion. Before fusion, univariate regression was conducted to remove sex and dummy‐coded site effects, as well as the top four principal components of the genomic SNP data to control for population stratification (Chen et al. 2019, 2022).

### 2.3 Estimation of Dynamic Functional Network Connectivity

#### 2.3.1 rsfMRI Data

The data we obtained contain rsfMRI scans of ~38 K individuals. UKB used identical scanner models, coils, software, and protocols across centers to ensure data harmonization as much as possible (Littlejohns et al. 2020). The rsfMRI scan parameters are as follows: resolution = 2.4 mm × 2.4 mm × 2.4 mm, matrix = 88 × 88 × 64, TE = 39 ms, TR = 735 ms, *α*= 51°, multiband factors = 8, duration = 6:10 min (Littlejohns et al. 2020). For the SZ data, detailed scanning parameters of each cohort can be found in Supporting Information S5.

#### 2.3.2 Preprocessing of rsfMRI

We downloaded the preprocessed UKB data, normalized the images to standard MNI space (http://www.mni.mcgill.ca/) and resampled to 3 mm × 3 mm × 3 mm voxels using the SPM nonlinear registration, followed by smoothing using a 6 mm FWHM Gaussian kernel. The rsfMRI data of the SZ cohorts were preprocessed using the fully automated NeuroMark pipeline (SPM12‐based) (Du et al. 2020; Fu et al. 2021) (see Supporting information S5 for details).

#### 2.3.3 Estimation of Windowed FNC

The Neuromark_fMRI_1.0 network template (Du et al. 2020; Fu et al. 2021) served as a reference in a spatial‐constrained independent component analysis (scICA) (Du and Fan 2013) to derive 53 subject‐level ICNs and their associated time courses (TCs). These 53 ICNs are functionally grouped into seven domains, including subcortical (SC), auditory (AUD), sensorimotor (SM), visual (VIS), cognitive control (CC), default mode (DMN), and cerebellum (CB). The subject‐level TCs then underwent additional processing steps, including detrending, despiking, regressions of the six realignment parameters and their temporal derivatives, and band‐pass filtering with 0.01–0.15 Hz. Windowed FNC was then estimated based on the processed TCs of 53 ICNs as described in our previous work (Allen et al. 2014; Du et al. 2020). In brief, a tapered sliding window (convolving a rectangular window of 20 TRs) was used to estimate covariance from the regularized precision matrix, resulting in a sequence of wFNCs of 1378 FNC pairs for each individual in the UKB and SZ cohorts.

#### 2.3.4 Estimation of dFNC States

The wFNCs of all UKB individuals were concatenated, on which K‐means clustering was applied to identify clusters of similar connectivity patterns across time windows and individuals, interpreted as dFNC states. In the current work, we identified four dynamic states, where the optimal number of states was determined as four based on the elbow method.

#### 2.3.5 Subject‐Level dFNC Features

For all the individuals in both UKB and SZ data, we assigned each wFNC to the nearest dFNC state based on its Euclidean distance to the state centroids. For each individual, wFNCs assigned to the same state were averaged to yield the most representative FNC patterns of this individual in a dynamic state, denoted as subject‐specific state‐average dFNC (sa‐dFNC) in the following text. In total, four sets of sa‐dFNCs were generated and used in the fusion analysis. As not all the individuals experienced all four states during the length of scan, sample sizes of dynamic fusion varied across states. Specifically, 31,814/32775/17929/27497 individuals were utilized for SNP fusion with states 1/2/3/4, respectively in UKB, and 633/786/648/449 individuals were used in the SZ data to validate the dFNC findings for each state. For each connectivity feature, regression was conducted to remove age, sex, and dummy‐coded site effects before fusion.

### 2.4 Dynamic Fusion

**FIGURE 1:**
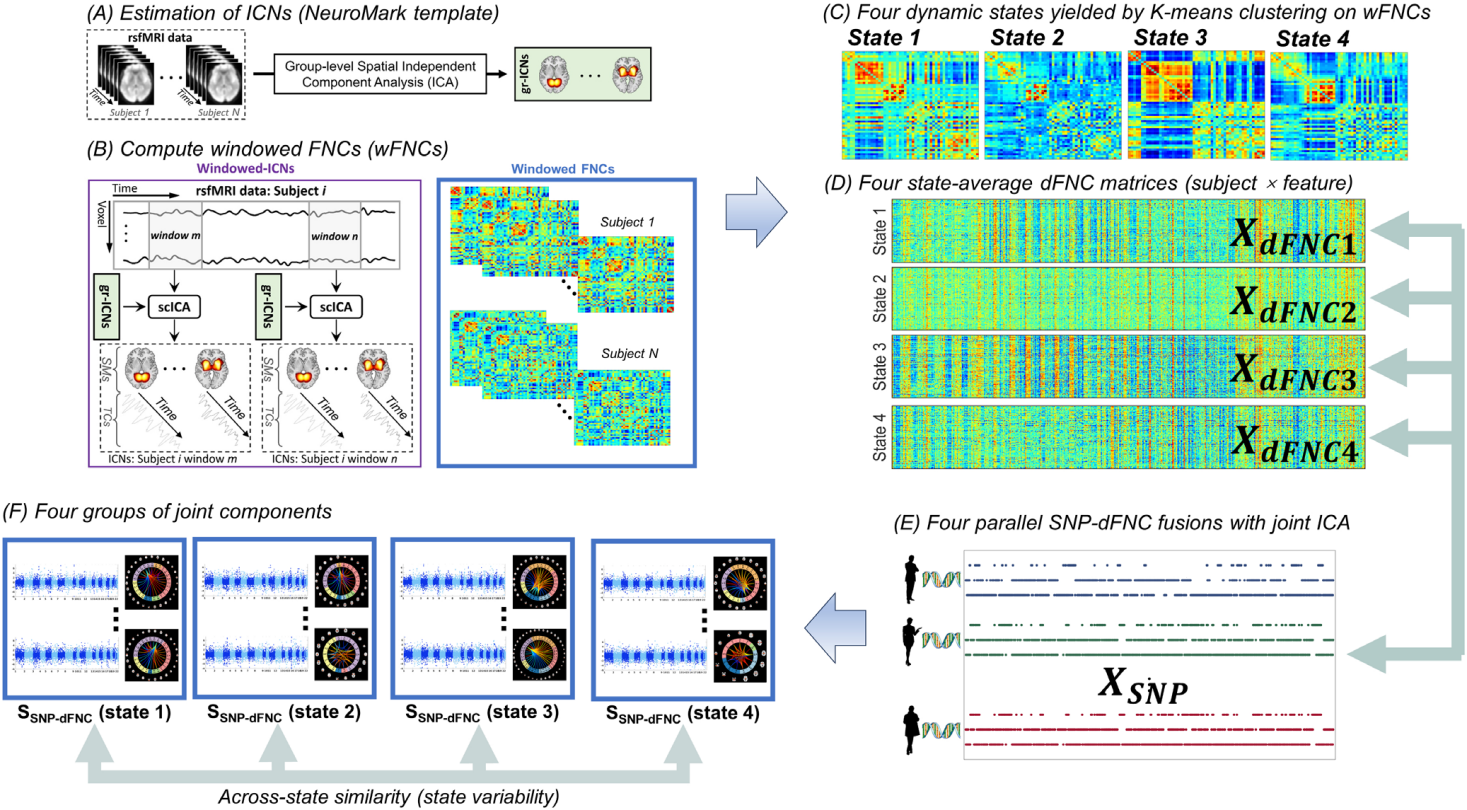
A diagram of dynamic fusion. The SNP data *X*_SNP_ was fused with the dFNC features of each state, for example, *X*_dFNC_ (State1), in parallel. *S*_SNP‐dFNC_ denotes the joint SNP‐dFNC components yielded by the dynamic fusion. Across‐state similarity was assessed between the four sets of components to indicate state variability for SNP and dFNC modalities, respectively. (A) Estimation of ICNs (NeuroMark template), (B) compute windowed FNCs (wFNCs), (C) four dynamic states yielded by K‐means clustering on wFNCs, (D) four stage‐average dFNC matrices (subject × feature), (E) four parallel SNP–dFNC fusions with jont ICA, and (F) four groups of joint components.

Figure 1 shows the diagram of dynamic fusion to link the same set of SZ risk SNPs separately with each sa‐dFNC matrix via jICA (Calhoun et al. 2007). JICA builds upon the Infomax algorithm, which achieves data decomposition by detecting features covarying with each other and clustering them into components following super‐Gaussian distributions (Bell and Sejnowski 1995). In this context, jICA captures joint SNP‐dFNC components that are maximally independent, with each component exhibiting similar SNP and dFNC variations across subjects. By decomposition *X* = *AS*, jICA decomposes the data *X* (i.e., combined data matrix of *X*_SNP_ and *X*_dFNC_) into a linear combination of a set of source components (*S*) and their associated loadings (*A*). S largely reflects how individual SNP and dFNC features are linearly weighted to collectively define an independent source, where heavily weighted features contribute more to component characteristics that differentiate from others. *A* reflects how source components are loaded on each subject. To identify dynamically tuned SNP manifolds, we conducted four parallel SNP‐dFNC fusions on the UKB data. For each fusion, the feature matrices (12,946 SNPs and 1378 connectivity pairs) were concatenated horizontally for jICA decomposition (Bell and Sejnowski 1995).

As there is currently no established rule for determining the optimal model order for our proposed dynamic fusion framework involving multiple parallel fusions, in this proof‐of‐concept study the model order was determined empirically based on the following considerations. First, we fixed the model order across the four parallel fusions to enable symmetric across‐state similarity evaluation and a fair comparison with the baseline approach without dynamic fusion. Second, we identified the knee point of the eigenvalue scree plot for each of the four sa‐dFNC matrices using the Elbow criterion. This yielded estimated model orders of 35, 40, 39, and 33, respectively (see Supporting Information S5 for the scree plots). Third, applying the same criterion to the SNP data suggested a comparable model order of 33. To balance across the data modalities, we considered the median of these five estimates, which resulted in a model order of 35. Fourth, we further examined the explained variance and found that a model order of 35 was the minimum required to capture at least 75% of the variance across all four dFNC states, while explaining approximately 41% of the variance in the SNP data. Based on these considerations, we fixed the model order at 35 for the dynamic fusion and conducted additional stability and validation analyses to further mitigate the risk of false positives.

With dynamic fusion, we obtained four sets of joint SNP‐dFNC components and included only super‐Gaussian components (see Supporting Information S5 for details) for further validation of SZ relevance as well as investigation into state variability of SNP manifolds. We conducted a three‐fold stability test, where the full sample was randomly partitioned into three subsets which were expected to be still powered for SNP‐dFNC fusion. The joint components extracted from the subsets were compared with those obtained from the full sample to assess the stability (measured by Pearson's correlation). To assess the influence of unequal sample sizes across four parallel fusions, we performed an additional analysis where the dynamic fusion analysis was repeated on a subset of 14,171 subjects shared across all four dynamic states, while keeping the same model order as in the original analysis. We also conducted unimodal ICA on the SNP data, as well as jICA on combined SNP and sa‐dFNC features of States 1 and 2 with similar sample sizes. These baseline results were compared with those of the dynamic fusion to examine the specificity of SNP manifolds.

### 2.5 Validation of SZ Relevance

With the prescreening, the SZ‐relevance of SNPs was grounded by the state‐of‐the‐art GWAS (Trubetskoy et al. 2022). In contrast, for the dFNC modality, we included whole‐brain connectivity features without prior disease‐relevance screening. Consequently, we specifically validated the dFNC elements of the identified joint components for SZ relevance in a fully independent dataset, with multiple comparisons controlled using FDR.

For this purpose, we first projected the dFNC elements onto the sa‐dFNC matrices of the independent SZ dataset that combined COBRE, FBIRN, and MPRC cohorts: $A^{\mathrm{SZ}}=X^{\mathrm{SZ}}{S_{\text{dFNC}}^{\mathrm{UKB}}}^{-1}$. The resulting loadings ($A_{\mathrm{SZ}}$) were then investigated for differences between individuals with SZ and controls using a two‐sample *t*‐test after regressing out age, sex, dummy‐coded site effects, and mean framewise displacement. Significant SZ‐discriminating dFNC elements were identified with FDR‐corrected *p* < 0.05 across all the four sets of elements. In the following text, the joint components with dFNC elements validated for SZ relevance will be termed as SZ‐relevant joint components. Only these SZ‐relevant joint components were carried forward for further interpretation.

### 2.6 Interpretation of Joint Components

First, we evaluated the components' across‐state similarity (Figure 1f). For each SNP‐dFNC component of a given state‐specific fusion, its most similar SNP and dFNC elements were identified in each of the other three fusions based on the correlations of *S*. The correlation‐based similarity measures were then averaged across the three most similar SNP and dFNC elements to reflect the overall across‐state similarity. Lower similarity indicates a multivariate SNP/dFNC factor shows more state variability, suggesting high state‐specificity.

Second, for all the SZ‐relevant components, we further assessed their dFNC associations with cognitive and symptom scores in the aggregated SZ data. Specifically, MATRICs Consensus Cognitive Battery (MCCB) scores were available for the COBRE cohort, and Computerized Multiphasic Interactive Neurocognitive System (CMINDS) scores were available for the FBIRN cohort. These two batteries are highly correlated, showing a linear relationship (Silverstein et al. 2010). We then normalized the MCCB and CMINDS scores in COBRE and FBIRN, respectively, and combined the normalized scores of the composite and six comparable domains (i.e., speed of processing, attention/vigilance, working memory, verbal learning, visual learning and reasoning/problem solving). Partial correlation was then assessed between the combined cognitive scores and dFNC elements (i.e., the projected loadings $A^{SZ}$), after regressing out age, sex, diagnosis, and dummy‐coded site effects. The sample size available for the cognitive association analysis was 335, 391, 333, and 220 for States 1–4, respectively. In parallel, we also assessed the partial correlations between dFNC and positive and negative subscales of the Positive and Negative Syndrome Scale (PANSS_Pos and PANSS_Neg) in the patient group, after regressing out age, sex, and dummy‐coded site effects. The sample size available was 178, 208, 154, and 114 for States 1–4, respectively.

Third, for all the SZ‐relevant joint components, interpretation focused on the top‐weighted SNPs and connectivity features within each component, as these features dominantly drove the component's characteristic covariation pattern identified by the algorithm, a strategy widely adopted in prior blind source separation studies (Chen et al. 2017, 2022; Calhoun et al. 2001; Xu et al. 2009). This step did not involve hypothesis testing and therefore we did not apply FDR correction. Specifically, the SNP and dFNC elements of SZ‐relevant joint components were, respectively, thresholded at |*z*‐score| > 3 (Chen et al. 2017, 2022; Calhoun et al. 2001; Xu et al. 2009) to identify heavily weighted top SNPs and connectivity features that were more tightly coupled with each other. The top connectivity pairs were then interpreted based on the involved ICNs. The top SNPs were annotated to genes and investigated for enrichment in biological processes using Gene Ontology (Thomas et al. 2022), where built‐in FDR correction was applied to control for multiple comparisons across tested pathways.

## 3 Results

We obtained four sets of joint SNP‐dFNC components from the dynamic parallel fusions, each set consisting of 35 joint components extracted from 12,946 SZ risk SNPs and 1378 sa‐dFNC features of one dynamic state, resulting in a total of 140 joint components. By projecting dFNC elements to the SZ cohort, 37 out of 140 joint components were validated as SZ‐relevant, as summarized in Table 1, where the percentage of explained variance (*r*; Glahn et al. 2007) ranged between 1.02% and 13.22%, reflecting the amount of variance explained in the SZ case–control difference. For each joint component identified from the fusion with one specific dynamic state, its most similar counterpart was identified among the remaining three fusions based on component similarity, measured using Pearson's correlation, as listed in Table 1.

**TABLE 1:** Summary of SZ‐relevant SNP‐dFNC components.

State	Comp	*r* ^2^	*p*	FDR q	State_similar^a^	Comp_similar^a^	SNP similarity^b^	dFNC similarity^b^
1	3	1.75%	8.45E−04	4.78E−03	4	23	0.2609	0.4679
1	6	2.23%	1.65E−04	1.54E−03	4	14	0.0718	0.3920
1	9	5.15%	7.65E−09	4.14E−07	3	7	0.3329	0.6698
1	10	1.28%	4.43E−03	1.73E−02	4	29	0.1537	0.6666
1	12	1.14%	7.14E−03	2.50E−02	3	18	0.3117	0.7797
1	13	2.07%	2.81E−04	2.18E−03	3	5	0.3507	0.7840
1	16	1.74%	8.67E−04	4.78E−03	4	17	0.0837	0.4094
1	19	2.05%	3.06E−04	2.25E−03	2	2	0.4234	0.6178
1	33	1.27%	4.58E−03	1.73E−02	2	33	0.9947	0.3474
2	2	1.15%	2.60E−03	1.21E−02	1	19	0.4234	0.6178
2	5	2.52%	7.64E−06	1.19E−04	4	20	0.1529	0.6263
2	9	1.49%	5.93E−04	3.96E−03	1	18	0.2544	0.6025
2	12	1.03%	4.36E−03	1.73E−02	1	14	0.1753	0.5037
2	22	4.13%	8.86E−09	4.14E−07	4	15	0.4315	0.8639
2	25	2.16%	3.49E−05	3.76E−04	1	20	0.3339	0.8042
2	30	1.16%	2.47E−03	1.19E−02	1	27	0.9804	0.2276
2	31	1.08%	3.58E−03	1.57E−02	1	34	0.9946	0.3256
2	32	2.33%	1.70E−05	2.16E−04	1	30	0.9995	0.5661
2	35	1.02%	4.57E−03	1.73E−02	1	35	0.9984	0.2991
3	2	3.85%	4.86E−07	9.71E−06	4	5	0.1353	0.6453
3	3	1.70%	8.87E−04	4.78E−03	4	9	0.1298	0.5990
3	4	2.99%	9.70E−06	1.36E−04	4	14	0.0823	0.5213
3	5	2.04%	2.64E−04	2.18E−03	1	13	0.3507	0.7840
3	7	13.22%	< 1.00E−16	< 1.00E−16	1	9	0.3329	0.6698
3	8	2.68%	2.81E−05	3.28E−04	2	11	0.2540	0.6009
3	9	2.33%	9.72E−05	9.72E−04	4	2	0.0204	0.5925
3	11	1.71%	8.39E−04	4.78E−03	4	12	0.1531	0.6006
3	15	3.91%	3.88E−07	9.05E−06	4	18	0.0738	0.6597
3	21	2.07%	2.41E−04	2.10E−03	1	22	0.1470	0.7591
3	22	4.08%	2.16E−07	6.05E−06	1	2	0.0605	0.5729
3	24	4.57%	3.85E−08	1.35E−06	1	8	0.1840	0.5620
3	29	1.24%	4.50E−03	1.73E−02	1	28	0.9152	0.2375
3	30	1.52%	1.68E−03	8.41E−03	2	32	0.9899	0.3389
3	31	1.21%	5.02E−03	1.85E−02	2	30	0.9382	0.1262
4	2	1.63%	6.81E−03	2.44E−02	3	9	0.0204	0.5925
4	15	2.35%	1.13E−03	5.87E−03	2	22	0.4315	0.8639
4	31	1.99%	2.74E−03	1.24E−02	2	33	0.9704	0.1464

^a^ State_similar and Comp_similar indicate the state and component index of the most similar counterpart.

^b^ SNP_similarity and dFNC_similarity report the correlations between the paired components for the SNP and dFNC elements, respectively.

The three‐fold stability test suggested that these joint components could be stably identified across three subsets, presenting similar patterns as those obtained from the full sample (mean correlation = 0.85, SD = 0.15). Additionally, when restricting the dynamic fusion to the 14,171 subjects shared across all four dynamic states, the resulting joint components also showed high consistency with those obtained in the original analysis. Specifically, the average correlation between the newly derived components and the original components was 0.89 (SD = 0.13). Among the 140 joint components, more than 83% exhibited correlations greater than 0.8. Importantly, the 37 SZ‐related joint components highlighted in Table 1 showed even stronger agreement, with an average correlation of 0.94 (SD = 0.05).

Table 2 summarizes the cognitive and clinical associations of these SZ‐relevant components. A total of seven cognitive measures (composite and six subdomains) and two PANSS subscales were tested. Among those showing associations with uncorrected *p* < 0.05, the strongest was reported in Table 2 to show the most relevant cognitive or symptom domain.

**TABLE 2:** Cognitive and symptom associations of SZ‐relevant SNP‐dFNC components.

State	Comp	Cognitive/clinical measure	*r*	*p*
1	3	VisualLearning	−0.1187	2.75E−02
1	9	VisualLearning	−0.1505	5.09E−03
1	12	VisualLearning	−0.1133	3.53E−02
2	9	AttentionVigilance	0.1275	1.12E−02
2	22	OverallCompositeScore	−0.1134	2.50E−02
2	32	PANSS_Neg	−0.1480	3.29E−02
3	4	ProcessingSpeed	0.1340	1.26E−02
3	7	ProcessingSpeed	−0.1528	4.39E−03
3	9	VisualLearning	0.1225	2.31E−02
3	11	VisualLearning	0.1149	3.32E−02
3	24	ReasoningProblemSolving	0.1101	4.10E−02
4	15	ReasoningProblemSolving	−0.1882	4.01E−03

The top SNPs and connectivity pairs of SZ‐relevant components were identified. Table S1 provides the list of the genes annotated to the top SNPs. The top connectivity pairs were characterized by the anatomical labels of the two involved ICNs, as summarized in Table S2. In addition, Manhattan and connectogram plots were generated for the SNP and dFNC elements, respectively (see Figures S1 and S2). Finally, for those SNP elements exhibiting significant enrichment in pathway analysis, we summarized the involved major biological processes in Table 3.

**TABLE 3:** Enriched biological processes identified from SZ‐relevant SNP‐dFNC components.

State	Comp	GO pathway	Fold enrichment	FDR *q*
1	3	Regulation of signaling (GO: 0023051)	2.29	2.02E−02
		Negative regulation of locomotion (GO: 0040013)	6.45	3.31E−02
		Neurogenesis (GO: 0022008)	3.31	3.39E−02
		Positive regulation of cell projection organization (GO: 0031346)	6.84	3.80E−02
		Neuron development (GO: 0048666)	3.94	3.83E−02
1	6	Neurogenesis (GO: 0022008)	3.91	1.92E−03
		Regulation of synaptic plasticity (GO: 0048167)	9.63	1.96E−02
1	9	Positive regulation of cell projection organization (GO: 0031346)	7.11	4.14E−03
		Regulation of synaptic plasticity (GO: 0048167)	10.45	4.43E−03
		Learning (GO: 0007612)	10.27	1.59E−02
		Memory (GO: 0007613)	10.9	4.70E−02
1	12	Positive regulation of cell projection organization (GO: 0031346)	8.89	8.05E−04
		Enzyme‐linked receptor protein signaling pathway (GO: 0007167)	5.37	4.06E−03
		Anatomical structure morphogenesis (GO: 0009653)	2.94	4.15E−03
		Taxis (GO: 0042330)	6.02	4.37E−03
		Locomotion (GO: 0040011)	5.8	4.45E−03
		Multicellular organism development (GO: 0007275)	2.31	4.60E−03
		Chemotaxis (GO: 0006935)	6.04	4.82E−03
		Regulation of neuron projection development (GO: 0010975)	6.36	5.05E−03
		Axon guidance (GO: 0007411)	8.56	1.61E−02
		Ephrin receptor signaling pathway (GO: 0048013)	22.88	2.16E−02
		Regulation of calcium ion transport (GO: 0051924)	7.61	2.30E−02
		Neurogenesis (GO: 0022008)	3.25	2.35E−02
1	13	Positive regulation of molecular function (GO: 0044093)	3.19	1.60E−02
		Synapse organization (GO: 0050808)	7.28	2.23E−02
		Regulation of neuron projection development (GO: 0010975)	5.38	2.95E−02
		Regulation of calcium ion transport (GO: 0051924)	7.37	3.07E−02
2	2	Regulation of intracellular signal transduction (GO: 1902531)	3.21	2.15E−03
		Learning or memory (GO: 0007611)	7.94	3.65E−03
		Positive regulation of cell projection organization (GO: 0031346)	5.63	3.24E−02
		Cardiac muscle cell contraction (GO: 0086003)	20.6	4.16E−02
		Regulation of long‐term synaptic depression (GO: 1900452)	41.52	4.49E−02
2	5	Organic substance transport (GO: 0071702)	2.78	7.51E−03
		Establishment of localization (GO: 0051234)	2.17	8.82E−03
		Regulation of monoatomic ion transport (GO: 0043269)	4.82	2.46E−02
2	9	Calcium ion import (GO: 0070509)	60.21	1.82E−02
2	22	Positive regulation of cell projection organization (GO: 0031346)	6.92	3.41E−02
2	25	Regulation of trans‐synaptic signaling (GO: 0099177)	6.44	3.08E−03
		Regulation of calcium ion transmembrane transport (GO: 1903169)	11.33	3.68E−03
		Learning (GO: 0007612)	10.03	3.34E−02
		Cardiac muscle cell action potential (GO: 0086001)	21.72	3.61E−02
3	2	Regulation of cell projection organization (GO: 0031344)	5.15	2.81E−02
3	4	Regulation of trans‐synaptic signaling (GO: 0099177)	6.32	3.28E−02
		Modulation of chemical synaptic transmission (GO: 0050804)	6.34	6.45E−02
3	5	Negative regulation of lyase activity (GO: 0051350)	41.18	2.37E−02
3	7	Cell–cell signaling (GO: 0007267)	3.97	1.66E−02
		System development (GO: 0048731)	2.37	2.68E−02
		Positive regulation of cell projection organization (GO: 0031346)	6.89	2.83E−02
		Multicellular organism development (GO: 0007275)	2.19	2.95E−02
3	8	Regulation of cell projection organization (GO: 0031344)	5.17	1.75E−03
		Calcium ion transmembrane transport (GO: 0070588)	8.42	2.46E−02
3	9	Regulation of synaptic plasticity (GO: 0048167)	10.6	2.06E−02
		Regulation of neuron projection development (GO: 0010975)	5.97	4.70E−02
		Regulation of heart rate by cardiac conduction (GO: 0086091)	29.12	4.75E−E−02
3	15	Action potential (GO: 0001508)	18.16	2.83E−03
		Cardiac muscle cell contraction (GO: 0086003)	29.56	6.33E−03
		Regulation of calcium ion transport (GO: 0051924)	7.73	1.91E−02
3	21	Regulation of trans‐synaptic signaling (GO: 0099177)	5.83	2.53E−02
		Regulation of adenylate cyclase activity (GO: 0045761)	21.45	2.73E−02
		Regulation of synaptic plasticity (GO: 0048167)	9.15	3.15E−02
		Glutamate receptor signaling pathway (GO: 0007215)	22.88	3.75E−02
		Calcium ion transmembrane transport (GO: 0070588)	8.38	3.80E−02
3	22	Cell–cell signaling (GO: 0007267)	3.96	1.69E−02
		Calcium ion transmembrane transport (GO: 0070588)	9.46	2.05E−02
		Atrial cardiac muscle cell action potential (GO: 0086014)	54.48	4.20E−02
3	24	Regulation of cell projection organization (GO: 0031344)	4.43	1.92E−02
		Anatomical structure morphogenesis (GO: 0009653)	2.32	4.94E−02
		Regulation of calcium‐mediated signaling (GO: 0050848)	12.68	5.01E−02
		Learning or memory (GO: 0007611)	5.96	5.04E−02
		Regulation of neuron projection development (GO: 0010975)	4.62	5.27E−02

**FIGURE 2:**
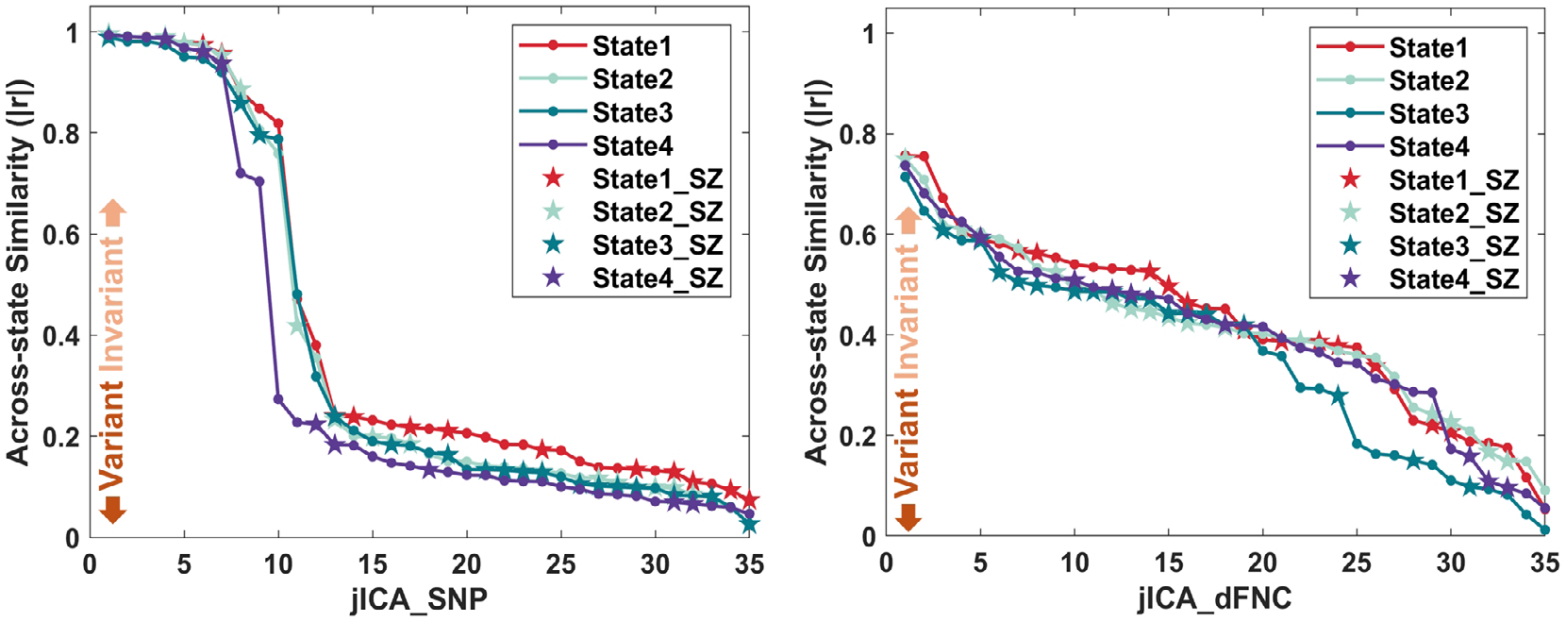
Across‐state similarity of SNP and dFNC elements respectively, where asterisks highlight SZ relevance.

Figure 2 shows the state variability of SNP and dFNC elements respectively, as measured by the average across‐state similarity and plotted in a sorted manner for each state. Note that for a joint component, its SNP and dFNC elements might present different levels of state variability. Overall, we observed a mixture of state‐invariant and state‐variant components for both SNP and dFNC modalities. The SZ‐relevant components were highlighted by asterisks, which did not appear to be enriched in the high or low end of state variability.

**FIGURE 3:**
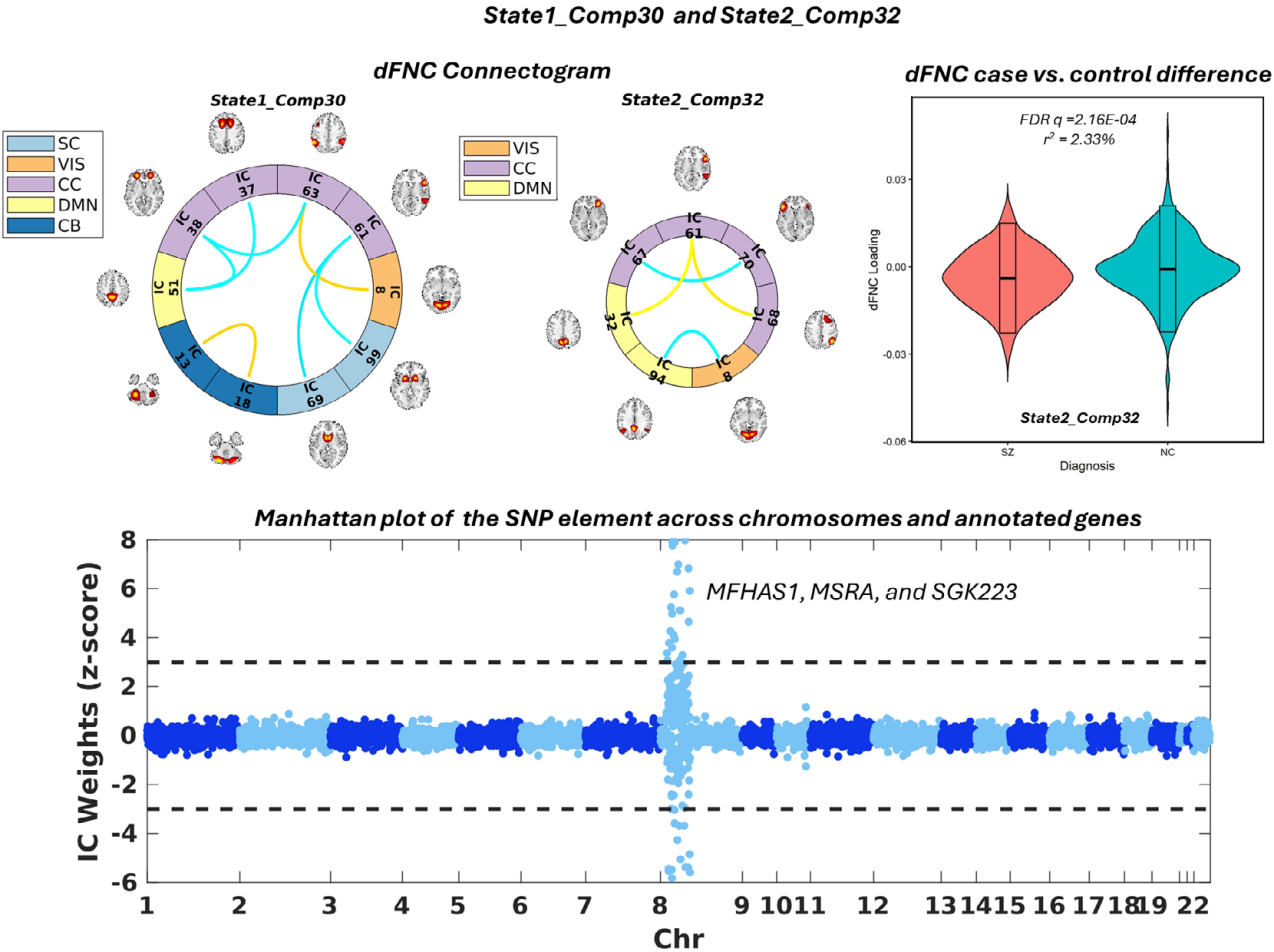
State1_Comp30 and State2_Comp32 are presented as an example pair of joint components with state‐invariant SNP elements. Top left: connectogram of State1_Comp30. Top middle: connectogram of State2_Comp32. Top right: violin plot showing the significant schizophrenia case–control difference observed in State2_Comp32. Bottom: Manhattan plot for State1_Comp30; dashed lines denote |*z*‐score| > 3.

For illustrative purposes, Figure 3 presents the 30th joint component of SNP‐dFNC_state1_ fusion (denoted as State1_Comp30), as well as State2_Comp32, serving as an example of two fusions yielding joint components with highly similar SNP elements (correlation = 1, as highlighted in Table 1). In Figure 3, we only show the Manhattan plot of the SNP element of State1_Comp30 to avoid redundancy. Thresholded at |*z*‐score| > 3, this SNP element was sparse and reflected the effects of a small set of top SNPs in Chromosome 8. The three annotated genes, *MFHAS1, MSRA*, and *SGK223*, were 100% overlapping between State1_Comp30 and State2_Comp32, with no significant pathway enrichment. Meanwhile, the dFNC elements of State1_Comp30 and State2_Comp32 showed an intermediate level of similarity. Despite not sharing top connectivity pairs, similar connections were noted between cognitive control and default mode network ICNs.

**FIGURE 4:**
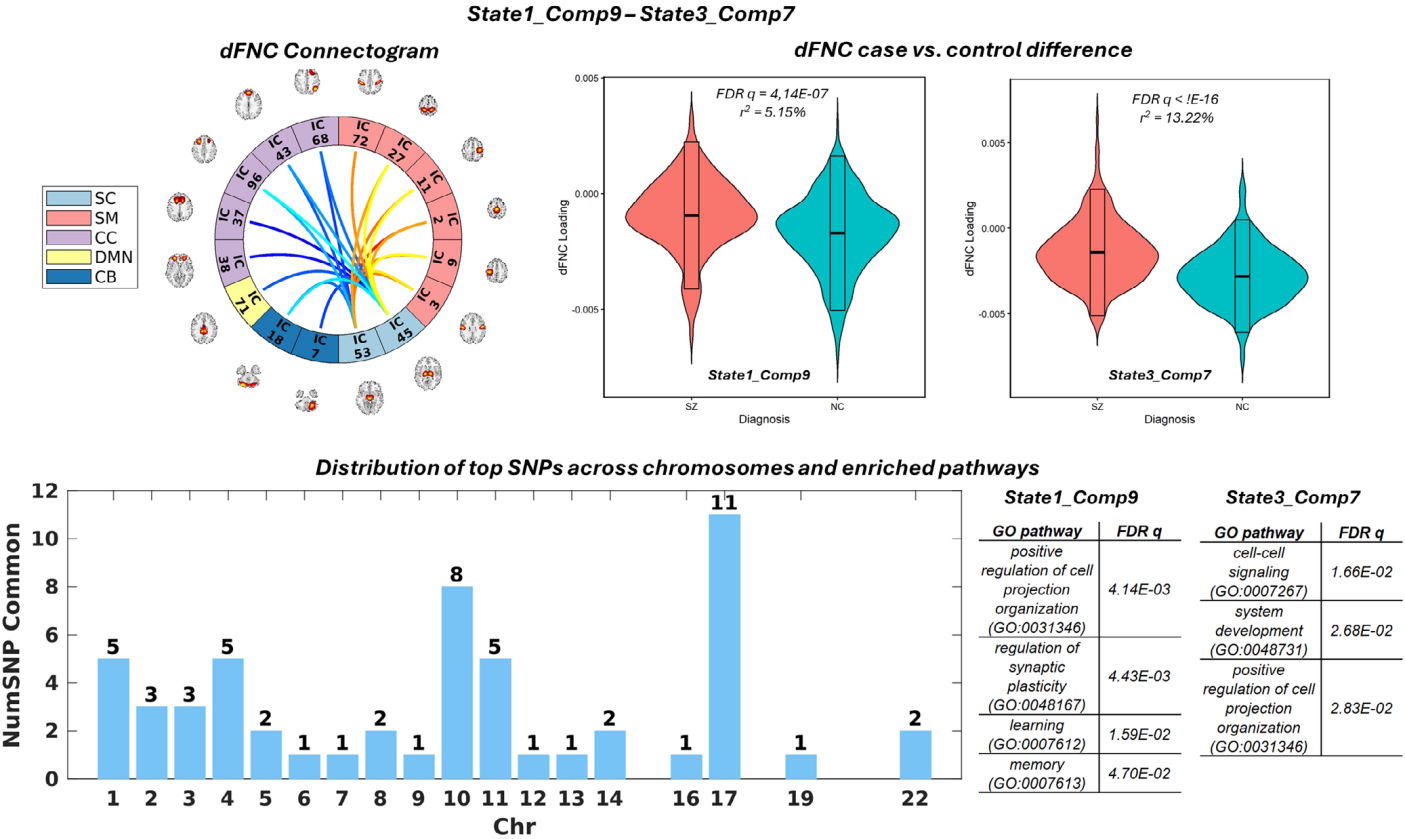
State1_Comp9 and State3_Comp7 are shown as an example pair of joint components with intermediate state variability. Top left: connectogram of top connectivity features shared by State1_Comp9 and State3_Comp7. Top middle and right: violin plots illustrating significant schizophrenia case–control differences for State1_Comp9 and State3_Comp7, respectively. Bottom: bar plot showing the distribution of top SNPs shared between the two components and the significantly enriched pathways associated with State1_Comp9 and State3_Comp7, respectively.

**FIGURE 5:**
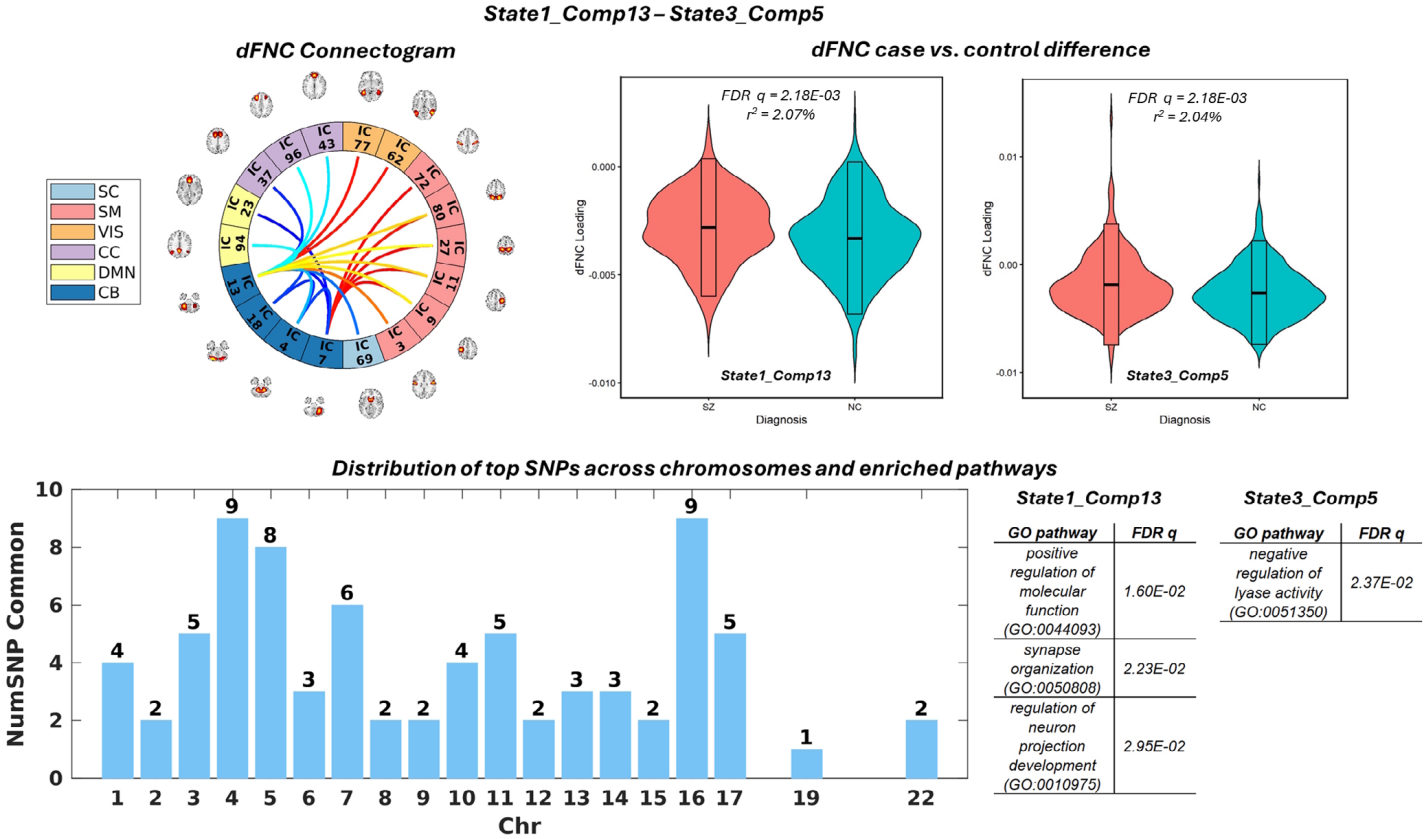
State1_Comp13 and State3_Comp5 are shown as another example pair of joint components with intermediate state variability. Top left: connectogram of top connectivity features shared by State1_Comp13 and State3_Comp5. Top middle and right: violin plots illustrating significant schizophrenia case–control differences for State1_Comp13 and State3_Comp5, respectively. Bottom: bar plot showing the distribution of top SNPs shared between the two components and the significantly enriched pathways associated with State1_Comp13 and State3_Comp5, respectively.

Figures 4 and 5 show two examples of parallel fusions yielding pairs of SZ‐relevant components of an intermediate level of similarity. Figure 4 presents the paired State1_Comp9 and State3_Comp7. The bar plot shows how the overlapping top SNPs were distributed across the genome. An overlapping ratio of 37% was noted between their annotated genes. As shown in Figure 4 and Table 3, both State1_Comp9 and State3_Comp7 were significantly enriched for the regulation of cell projection organization. Other enriched pathways included synaptic plasticity, learning, and memory for State1_Comp9, as well as cell signaling and system development for State3_Comp7. Figure 4 also shows the connectogram of the overlapping top connectivity features, where warm and cool color codes indicate positive and negative joint component weights, respectively. State1_Comp9 and State3_Comp7 showed similar thalamus‐ and subthalamus/hypothalamus‐hub connections to other ICNs (see Table S2 for labels of the ICNs). Along with the significant case–control differences (Table 1 and Figure 4), State1_Comp9 and State3_Comp7 suggest increased thalamus connections to sensorimotor networks, as well as decreased thalamus connections to cognitive control, default mode, and cerebellar networks in individuals with SZ compared to controls.

State1_Comp13 and State3_Comp5 make another pair of components showing an intermediate level of state variability, as presented in Figure 5. The distribution of overlapping top SNPs is shown in the bar plot, resulting in an overlapping ratio of 31% between the annotated genes. At the genetic pathway level, State1_Comp13 was significantly enriched for neuron projection, synapse organization, and calcium ion transport, while State3_Comp5 was enriched for the regulation of lyase activity. Both State1_Comp13 and State3_Comp5 showed significant case–control differences and highlighted cerebellum‐hub connections to other ICNs, together pointing to SZ‐related increased cerebellum connections to visual and sensorimotor networks, as well as decreased cerebellum connections to subcortical, cognitive control, and default mode networks.

**FIGURE 6:**
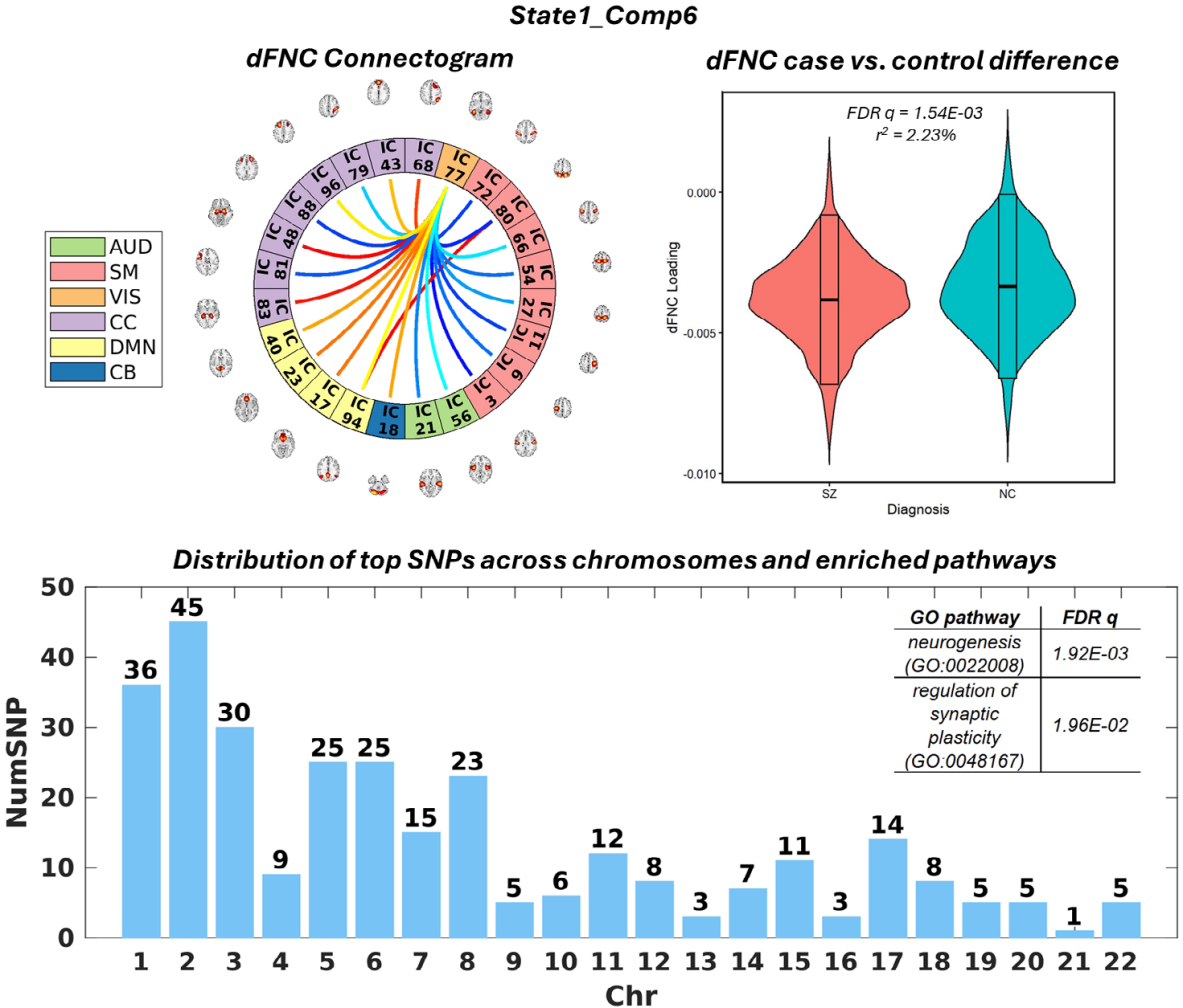
State1_Comp6 is shown as an example of a joint component with high state variability. Top left: Connectogram of State1_Comp6. Top right: violin plot illustrating the significant schizophrenia case‐control difference in State1_Comp6. Bottom: bar plot showing the distribution of top SNPs and significantly enriched pathways associated with State1_Comp6.

**FIGURE 7:**
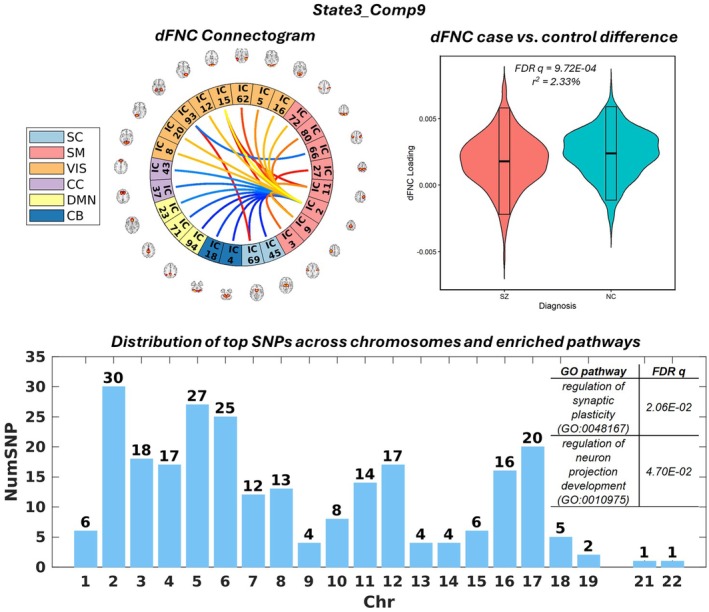
State3_Comp9 is shown as another example of a joint component with high state variability. Top left: connectogram of State3_Comp9. Top right: violin plot showing the significant schizophrenia case–control difference in State3_Comp9. Bottom: bar plot showing the distribution of top SNPs and the significantly enriched pathways associated with State3_Comp9.

Finally, Figures 6 and 7 present State1_Comp6 and State3_Comp9, respectively, as two examples showing a high level of state variability and therefore considered as state‐variant manifolds. As shown in Figure 6, State1_Comp6 captured the connections of middle temporal gyrus (MTG), labeled as a visual network, to other ICNs. The case–control difference largely indicates decreased MTG connections to cerebellum, default mode, and cognitive control networks, as well as increased MTG connections to auditory and sensorimotor networks in SZ. The SNP element of State1_Comp6 was significantly enriched for regulation of synaptic plasticity and neurogenesis. For State3_Comp9, its SNP element was significantly enriched for synaptic plasticity and neuron projection development, as shown in Figure 7. The connectogram, along with the case–control difference, largely indicated decreased sensorimotor connections to visual networks, as well as increased sensorimotor connections to cerebellum, subcortical, default mode, and cognitive control networks in SZ.

Notably, the parallel dynamic fusions yielded different SNP components compared to applying unimodal ICA on the SNP data. Specifically, 9 out of 35 unimodal SNP components were able to find counterparts in dynamic fusion with correlation > 0.85, echoing the aforementioned state‐invariant components. For the remaining unimodal SNP components, the similarity with dynamic fusion ranged between 0.05 and 0.72 with a mean of 0.19. Similarly, when the SNPs were fused with sa‐dFNC features of both States 1 and 2, 5 out of 35 components found counterparts, with the remaining similarity ranging between 0.08 and 0.79 with a mean of 0.45. These observations indicated that locally optimizable SNP factors might be discovered by separately linking SNPs to each dFNC state.

## 4 Discussion

As expected, both SNP and dFNC elements yielded by the dynamic fusion showed a wide range of state variability, while differences were also noted between the two modalities. As shown in Figure 2, the SNP modality reached higher across‐state similarity while more dFNC elements presented an intermediate level of state variability. The highly similar, or state‐invariant SNP elements were in general accompanied by dFNC elements with intermediate to low similarity (Table 1), and typically highlighted a small set of top genes and connectivity pairs, as shown in Figure 3 for an example. We interpret this type of SNP‐dFNC associations as localized or regional genetic variants affecting different small sets of connectivity pairs in different states.

Across four parallel fusions, a total of 37 dFNC elements of the joint components showed significant differences between SZ cases and controls in the aggregated independent cohorts. These 37 joint components were considered SZ‐relevant, overall evenly distributed across the range of state variability, as shown in Table 1 and Figure 2, indicating no bias in high or low state variability for SZ relevance.

State3_Comp 7 was paired with State1_Comp 9 as an example of a component showing an intermediate level of state variability, which happened to be the top two SZ‐discriminating components, explaining 13.22% and 5.15% of the variance in the case–control difference respectively. Both of their dFNC elements, as shown in Figure 4, highlighted a subset of the connectome which could be described as thalamus‐ and subthalamus/hypothalamus‐hub connections to other ICNs. Thalamus plays a key role in information processing, and the abnormalities in the frontal‐thalamic‐cerebellar circuitry are involved in a wide range of symptoms, including SZ (Andreasen 1997; Pergola et al. 2015). Thalamic functional dysconnectivity has also been documented in SZ (Gong et al. 2019; Giraldo‐Chica et al. 2018; Ferri et al. 2018). Our findings echo previous work to confirm disrupted thalamic connections in SZ, and further reveal a thalamus‐hub connectivity pattern that existed in multiple dynamic states, likely reflecting a primary composition of the connectome.

The linked genomic factors of State1_Comp9 and State3_Comp7 shared 37% of the annotated genes, both enriched for regulation of cell projection organization, a biological process that results in the assembly, arrangement of constituent parts, or disassembly of a prolongation or process extending from a cell, for example, a flagellum or axon, as defined by Gene Ontology. This raises the question of whether the disrupted cell projection organization may contribute to functional dysconnectivity, which requires the integration of multiscale data resources to verify and understand how it happens. State1_Comp9 was also enriched for the regulation of synaptic plasticity, as well as learning and memory. There has been the hypothesis of synaptic dysfunction in SZ (Frankle et al. 2003; Stephan et al. 2006). Regional decrease in the density of postsynaptic elements has been reported for SZ in a meta‐analysis of postmortem brain studies (Berdenis van Berlekom et al. 2020). The current finding also finds support from previous work where reduced thalamic‐prefrontal connectivity was associated with impaired working memory in SZ (Giraldo‐Chica et al. 2018). While in‐depth functional annotation is beyond the scope of the current study, our observation encourages further investigations at the cellular level on how the identified genomic risk translates into functional dysconnectivity between the thalamus and other brain networks that are characteristic of SZ.

Another pair of SZ‐relevant components with an intermediate level of variability included State1_Comp13 and State3_Comp5, both of which highlighted cerebellum‐hub connections to other ICNs (Figure 5), explaining 2.07% and 2.04% variances in case–control differences, respectively. Also, as an important region of the frontal‐thalamo‐cerebellar circuitry, the role of the cerebellum in cognitive symptoms of SZ has been more and more emphasized (Andreasen et al. 1996; Ide and Chiang‐shan 2011; Schmahmann et al. 2019). More recently, gray matter reduction in the cerebellum has been suggested to modulate static and dynamic cerebellum dysconnections with the thalamus and frontal regions in SZ (He et al. 2019). Our findings of the cerebellum‐hub connectivity pattern, along with the thalamus‐hub components discussed above, are in high concert with the literature to emphasize the key hub roles of these two regions in SZ‐related dysconnectivity. The linked genomic factors of these two pairs shared 31% of the annotated genes, although enriched in different biological processes. It is noted that in addition to synapse organization, whose relevance to SZ has been discussed above, State1_Comp13 was enriched for regulation of neuron projection development, which also plays an important role in the neuropathology of SZ. Neuron projection may refer to any process extending from a neural cell, such as axons or dendrites, that are involved in communication between neurons and hence functional connectivity. Again, despite that these enriched biological processes may be reasonably linked to the dFNC features, further verification is warranted and investigations are needed to characterize whether genomic risk affects the brain through regulating gene expression or other mechanisms.

State1_Comp30 and State2_Comp32 are examples of state‐invariant localized SNP elements, which showed a high correlation close to 1 (Table 1). Their SNP elements highlighted the same annotated genes: *MFHAS1*, *MSRA*, and *SGK223* (Figure 3 and Table S1). The linked dFNC elements reflected connections between visual, cognitive control, and default mode networks (Figure 3). It is noted that *SGK223* (also known as *PRAG1* or pragmin) induces Rho‐dependent cell contraction in neuronal cells and may regulate neurite outgrowth (Tanaka et al. 2006). It remains to be elucidated whether any additional pathways may bridge between these genes and connectivity features.

Last but not least, we have identified joint components located at the high end of state variability, such as State1_Comp6 (Figure 6) and State3_Comp9 (Figure 7), which could likely be elicited only upon dynamic fusion. And we interpret them as state‐variant manifolds. Note that State1_Comp6, State1_Comp9, and State3_Comp9 were all enriched for synaptic plasticity even though they were not correlated with one another; the top SNPs showed a low average overlap of 0.18. This observation indicates that the State1_Comp6 and State3_Comp9 SNPs may complement State1_Comp9 to provide a more complete picture of the genomic factor that confers risk to SZ by affecting synaptic plasticity. This also presents the benefit of dynamic fusion to augment the SNP factors across state manifolds that may provide additional insights into the underlying biology. State1_Comp6 describes an association between MTG‐hub connections and a genomic factor enriched for the regulation of synaptic plasticity. Both structural abnormalities in MTG and MTG dysconnectivity have been linked to auditory verbal hallucinations in SZ (Cui et al. 2018; Zhang et al. 2017). State3_Comp9 highlights sensorimotor‐hub connections, and the disintegration of sensorimotor networks has also been well documented in SZ (Kaufmann et al. 2015). Synaptic plasticity is a key biological process suggested to underlie brain connectivity (Stampanoni Bassi et al. 2019). Consequently, it is reasonable that several of our identified SNP elements were enriched for this biological process.

While these SNP elements did not significantly overlap but all enriched for pathways related to synaptic plasticity, it is intriguing to investigate whether distinct genetic variants may converge on the same high‐level biological pathway while influencing different cell types, regions, or brain circuitry, which may in turn manifest as associations with different dFNC components across dynamic states. For example, one gene uniquely highlighted for State1_Comp9, *SORCS3*, has been reported to exhibit strong early expression in thalamic nuclei during development (Oetjen et al. 2014), suggesting potential regional specificity. Another gene associated with the same component, *GRIN2A*, is known to participate in thalamocortical signaling (Stedehouder et al. 2026; Myers et al. 2019), pointing to potential circuitry‐level specificity. These observations suggest that genetic influences on synaptic plasticity may operate through region‐ or circuitry‐specific mechanisms, potentially affecting the maturation or regulation of thalamocortical pathways that are highlighted in particular dynamic connectivity states.

A total of 12 out of 37 SZ‐relevant components were associated with cognitive measures or symptom scores. Despite no FDR correction being imposed, the observed cognitive or symptom associations as summarized in Table 2 were all consistent with the observed disease effects, that is, the hyper‐ or hypoconnectivity patterns noted in the SZ group were also related to higher symptom scores or poorer cognitive performance. These SZ‐relevant components appeared to show stronger associations with cognitive measures than symptom scores, which might be due to the differences in available sample sizes, and this observation awaits further verification. In the meantime, the most associated cognitive measures of these components spanned various cognitive domains as well as composite scores. Overall, these findings suggest potential pathways linking genetic risk disrupting key biological processes (e.g., regulation of synaptic plasticity, cell projection, memory) to dysconnectivity in large intrinsic functional networks and cognitive impairment, motivating further mechanistic investigations.

This work presents a novel framework and some initial evidence to showcase how dynamic SNP‐dFNC fusion expands the SNP modality for biologically meaningful state‐variant manifolds that offer additional insights into SZ. The results should be interpreted in light of the following limitations. First, a more sophisticated adaptive normalization may be needed to better compensate for the difference in SNP and dFNC data structure so that more balanced data decomposition can be achieved to allow both modalities contributing equally to the components (Khalilullah et al. 2023). Second, the sa‐dFNC matrix was generated based on hard clustering where each windowed FNC was exclusively assigned to one dFNC state. This led to unequal sample sizes across four dynamic states. The potential influence of unbalanced sample sizes was assessed with stability tests and additional analyses on 14,171 subjects shared across four states. Overall, the results suggest that our main findings and interpretations are largely robust to the differences in sample sizes, and that the components uniquely observed within state‐specific fusions are more likely to reflect state‐specific patterns rather than artifactual variability driven by unbalanced sample sizes across four states. That said, we plan to explore fuzzy clustering or meta‐state approaches to address this limitation. These approaches allow each dFNC time window to have a probabilistic rather than categorical state membership, or represent a linear combination of dynamic states rather than being assigned to one state (Miller et al. 2016; Ellis et al. 2023), which facilitates a more flexible characterization of temporal dynamics. Third, the current SNP‐dFNC dynamic fusion utilized the large population cohort of UKB for discovery. Although we validated the findings in the combined SZ cohort, it remains a question of whether a dynamic fusion directly applied to SZ cohorts may yield additional insights. Fourth, we did not strictly calibrate the window lengths of UKB and SZ data in the dFNC analysis. That said, the dynamic fusion framework appears to robustly identify meaningful components that could be validated for disease relevance. Fifth, analytical decisions made at different stages, such as preprocessing strategies and model parameterization, may potentially influence the results, which are inherent limitations associated with a complex, multimodal data‐driven framework. Therefore, our findings should be interpreted within this context. Continued validation across independent datasets and complementary methodological frameworks will be important for strengthening the robustness and generalizability of the reported results.

In conclusion, this work aims to present a novel dynamic fusion framework to identify state‐variant manifolds of SNP‐dFNC associations for additional biological insights. Overall, results support the hypothesis that dynamic fusion yields meaningful state‐variant manifolds. Particularly, the SNP manifolds uniquely elicited by the dynamic fusion complement each other in terms of biological interpretation. This key finding suggests that genetic influences on the brain may operate through cell‐type‐, region‐, or circuitry‐specific mechanisms, motivating further investigation into heterogeneous molecular pathways underlying dynamic brain connectivity. In this context, the proposed dynamic fusion framework provides a unique lens to uncover unique SNP correlates otherwise unseen, promising additional mechanistic insights into disease pathology.

## Author Contributions

J.C. and V.D.C. designed the study. Z.F. contributed to the data preprocessing. J.C. conducted the analyses and drafted the paper with feedback from all authors. All authors contributed to the writing process and have approved the final manuscript.

## Funding

This work was supported by the National Institutes of Health (R01EB005846, R01EB006841, R01MH094524, R01MH106655, and R01MH136665) and the National Science Foundation (1539067).

## Conflicts of Interest

The authors declare no conflicts of interest.

## Supporting information


**Figure S1:** Connectogram plot of the top connectivity pairs for each of the identified schizophrenia‐relevant components.

**Figure S2:** Manhattan plot of the top SNPs for each of the identified schizophrenia relevant components.

**Table S1:** Genes annotated to the top SNPs of each schizophrenia‐relevant component.

**Table S2:** Intrinsic connecitivity network labels of the top connectivity pairs for each schizophrenia‐relevant component.

**Supporting Information S5: Text 1** Resting‐state fMRI data collection.
**Supporting Information S5: Text 2** Resting‐state fMRI data preprocessing.
**Supporting Information S5: Text 3** We identified the knee point of the eigenvalue scree plot for each of the four state‐average dFNC matrices using the Elbow criterion.
**Supporting Information S5: Text 4** Identify super‐Gaussian components for further investigation.

## Data Availability

The datasets analyzed in this study include the UK Biobank (UKB) and the COBRE dataset, both of which are fully publicly accessible through their respective data access platforms. The FBIRN and MPRC datasets are not fully publicly available but can be obtained upon reasonable request from the corresponding authors and the respective principal investigators, subject to institutional approvals and data use agreements.
