# Supplementary material for: Dynamic Fusion of Genomics and Functional Network Connectivity in UK Biobank Reveals Schizophrenia‐Related SNP Manifolds
Source: Hum Brain Mapp. 2026 Apr 20;47(6):e70530. doi: 10.1002/hbm.70530 (PMC13095861; doi:10.1002/hbm.70530)
Supplement: Supplementary file 1 — Figure S1: Connectogram plot of the top connectivity pairs for each of the identified schizophrenia‐relevant components. [file HBM-47-e70530-s001.pdf]

Figure S1: Connectogram plot of the top connectivity pairs for each of the identified schizophrenia-relevant components

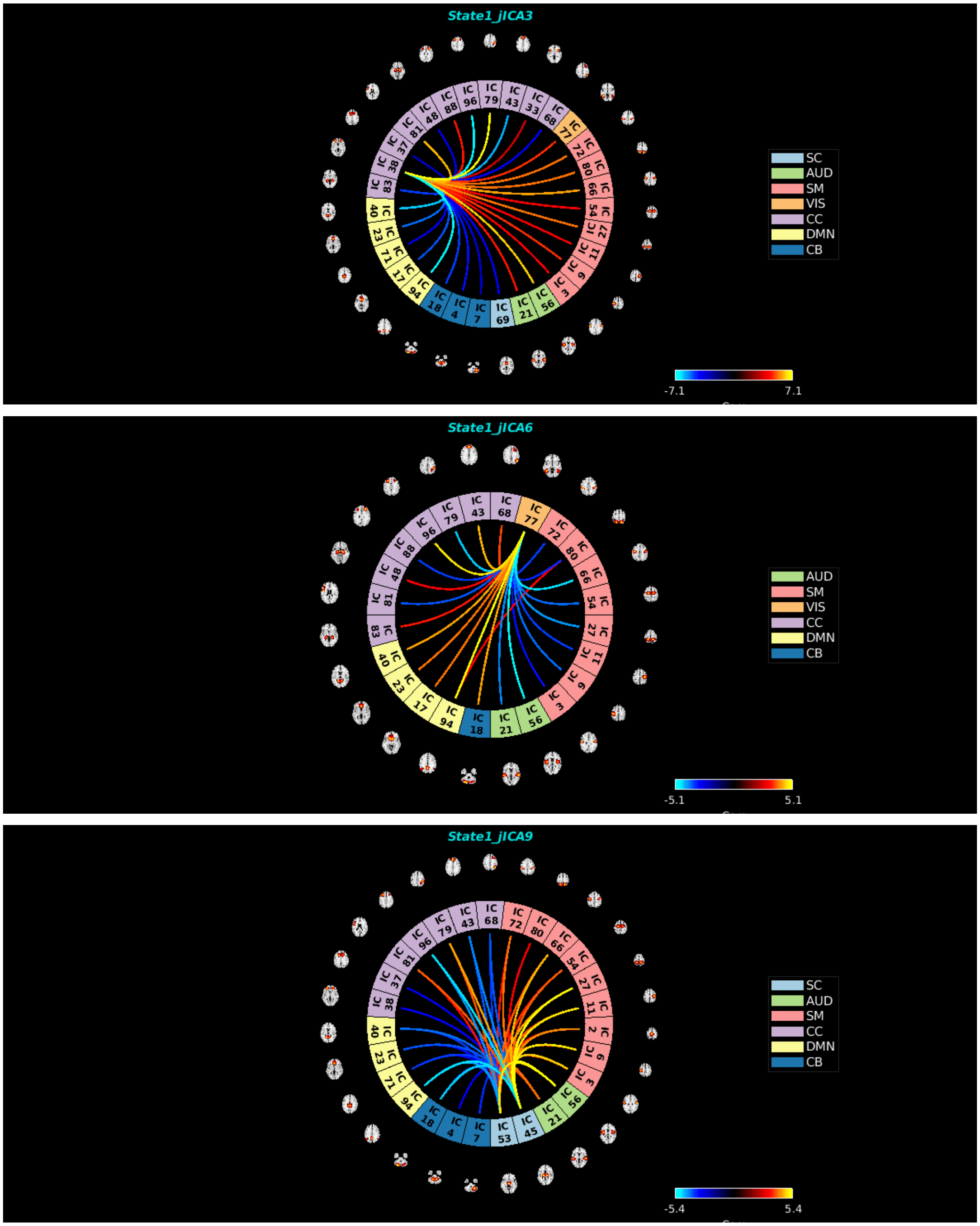

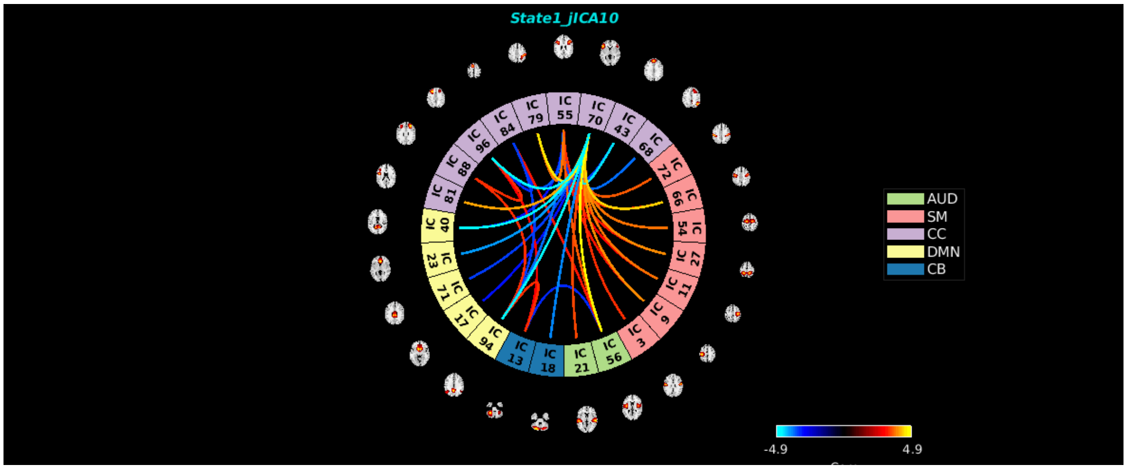
State1_jICA10
AUD
SM
CC
DMN
CB
-4.9
4.9

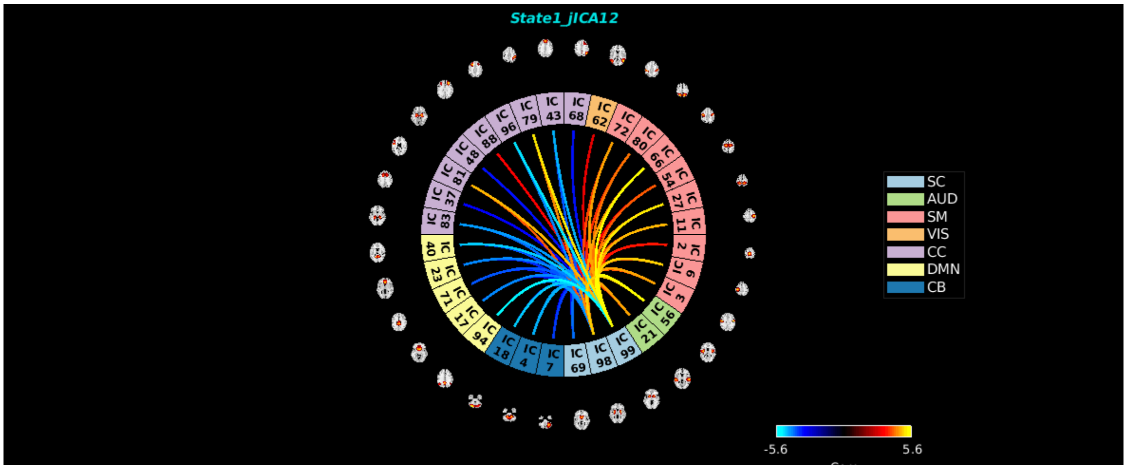
State1_jICA12
SC
AUD
SM
VIS
CC
DMN
CB
-5.6
5.6

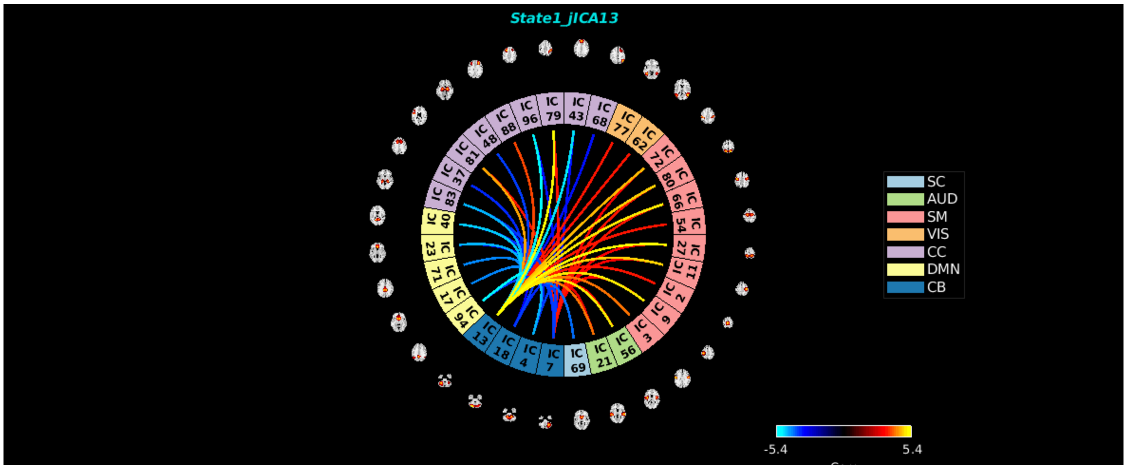
State1_jICA13
SC
AUD
SM
VIS
CC
DMN
CB
-5.4
5.4

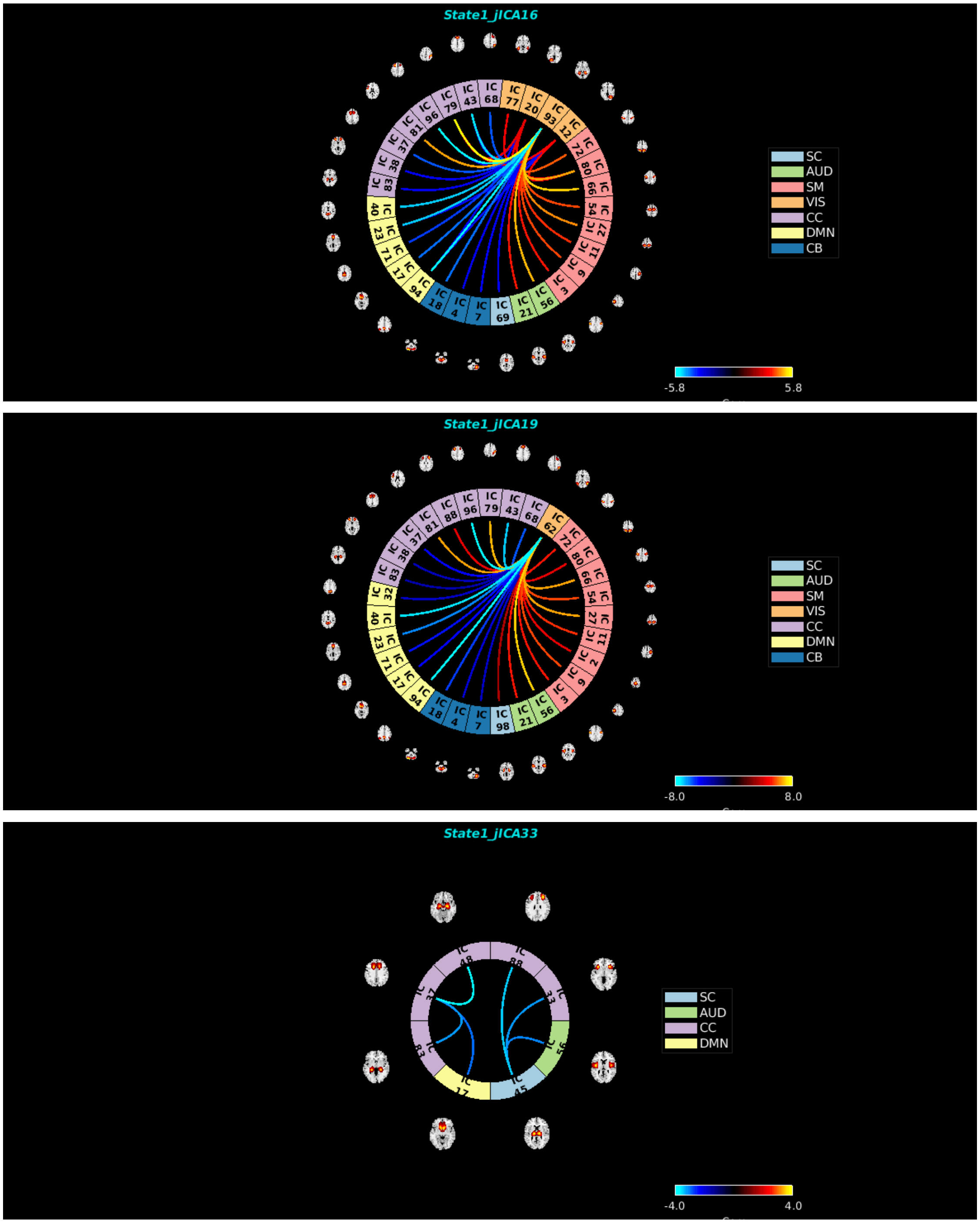
State1_jICA16
SC
AUD
SM
VIS
CC
DMN
CB
-5.8
5.8
State1_jICA19
SC
AUD
SM
VIS
CC
DMN
CB
-8.0
8.0
State1_jICA33
SC
AUD
CC
DMN
-4.0
4.0

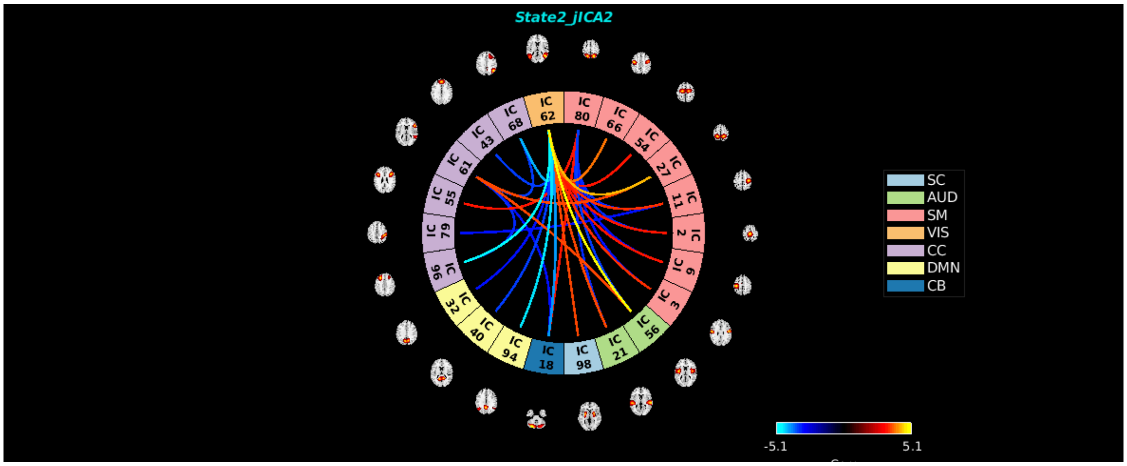
State2_jICA2
IC 62
IC 80
IC 66
IC 54
IC 27
IC 11
IC 2
IC 9
IC 3
IC 56
IC 21
IC 98
IC 18
IC 94
IC 40
IC 32
IC 96
IC 79
IC 55
IC 61
IC 43
IC 68
SC
AUD
SM
VIS
CC
DMN
CB
-5.1
5.1

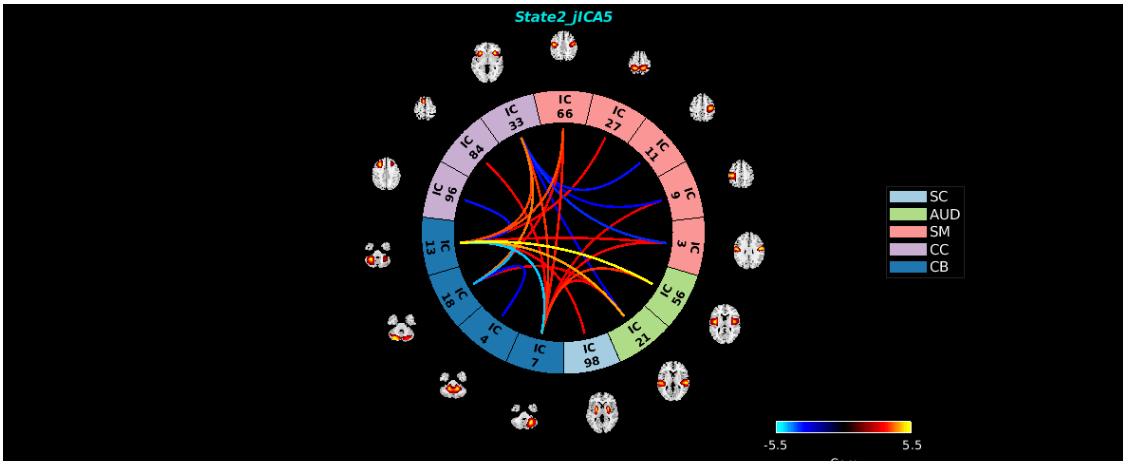
State2_jICA5
IC 66
IC 27
IC 11
IC 9
IC 3
IC 56
IC 21
IC 98
IC 7
IC 4
IC 18
IC 13
IC 96
IC 84
IC 33
SC
AUD
SM
CC
CB
-5.5
5.5

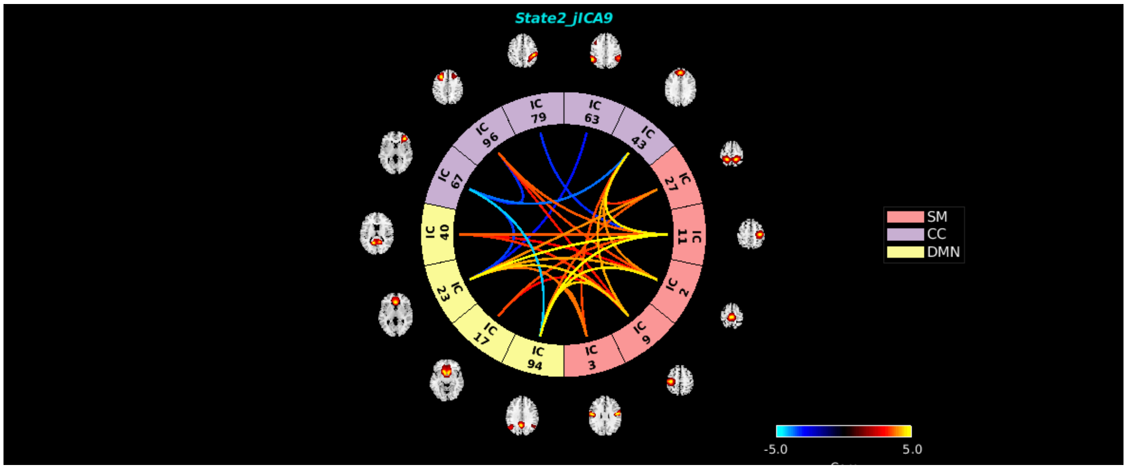
State2_jICA9
IC 63
IC 43
IC 27
IC 11
IC 2
IC 9
IC 3
IC 94
IC 17
IC 23
IC 40
IC 67
IC 96
IC 79
SM
CC
DMN
-5.0
5.0

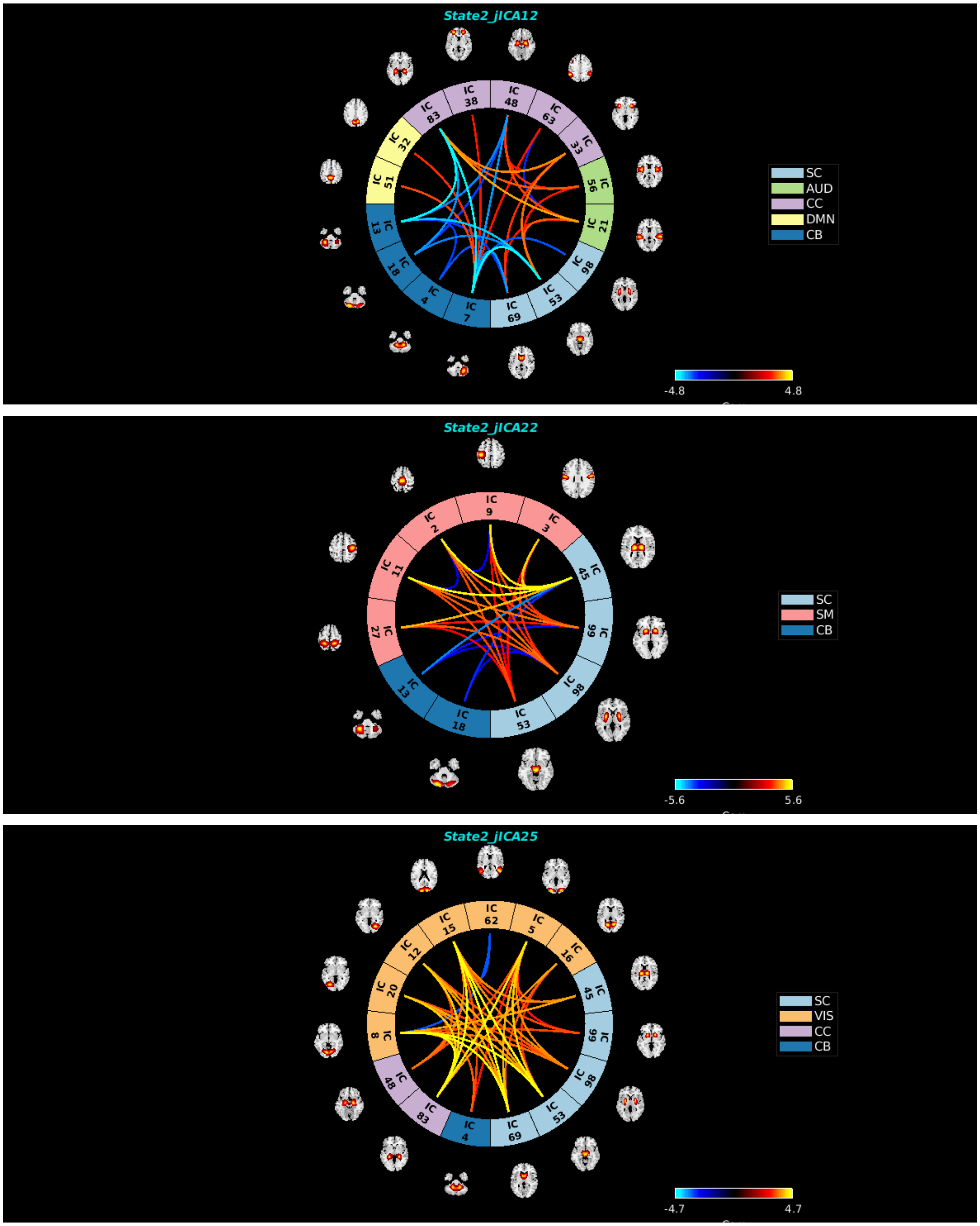
State2_jICA12
SC
AUD
CC
DMN
CB
-4.8
4.8
State2_jICA22
SC
SM
CB
-5.6
5.6
State2_jICA25
SC
VIS
CC
CB
-4.7
4.7

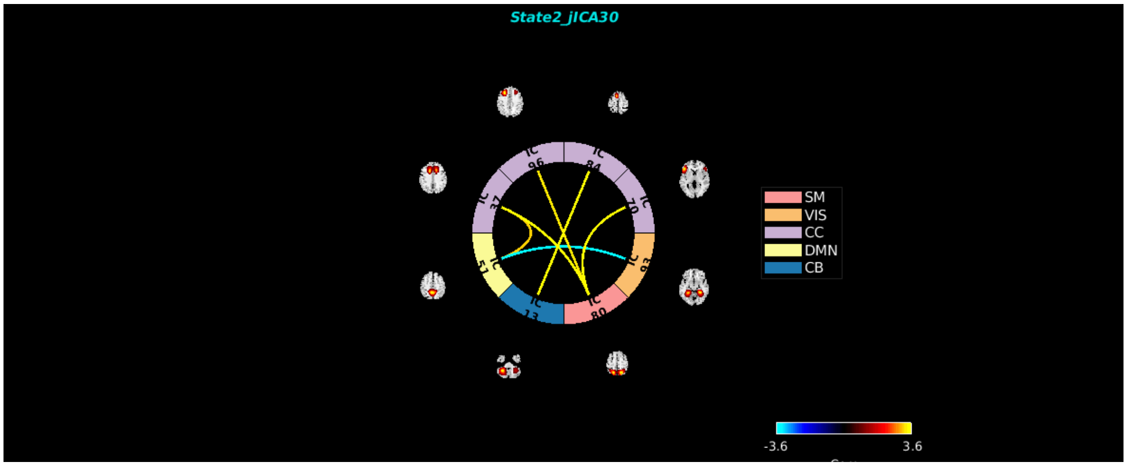
State2_jICA30
IC 96
IC 84
IC 70
IC 63
IC 80
IC 13
IC 51
IC 37
SM
VIS
CC
DMN
CB
-3.6
3.6

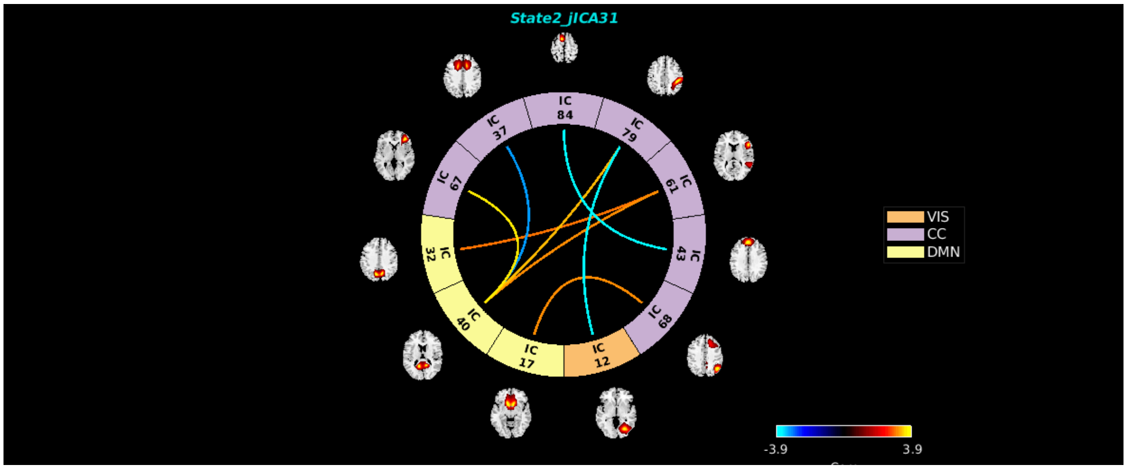
State2_jICA31
IC 84
IC 79
IC 61
IC 43
IC 68
IC 12
IC 17
IC 40
IC 32
IC 67
IC 37
VIS
CC
DMN
-3.9
3.9

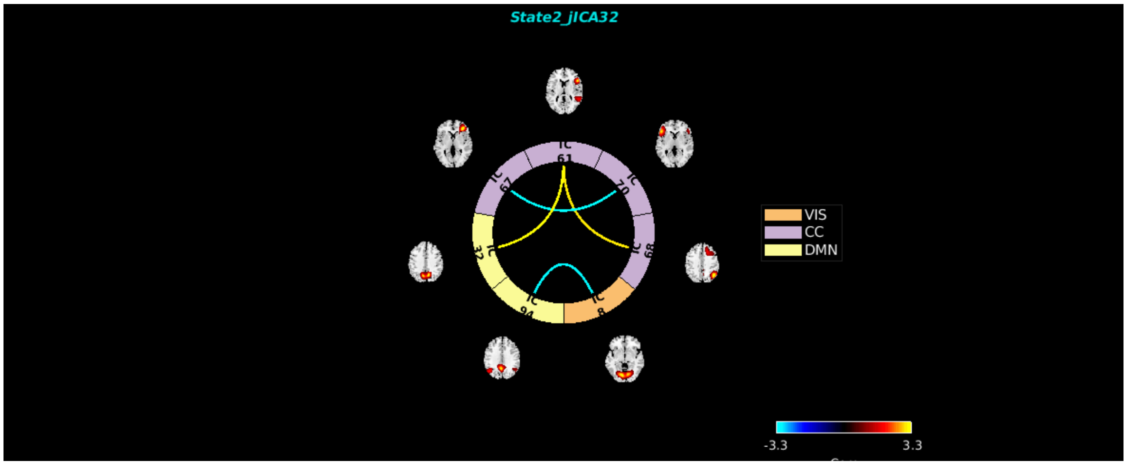
State2_jICA32
IC 61
IC 70
IC 68
IC 8
IC 94
IC 32
IC 67
VIS
CC
DMN
-3.3
3.3

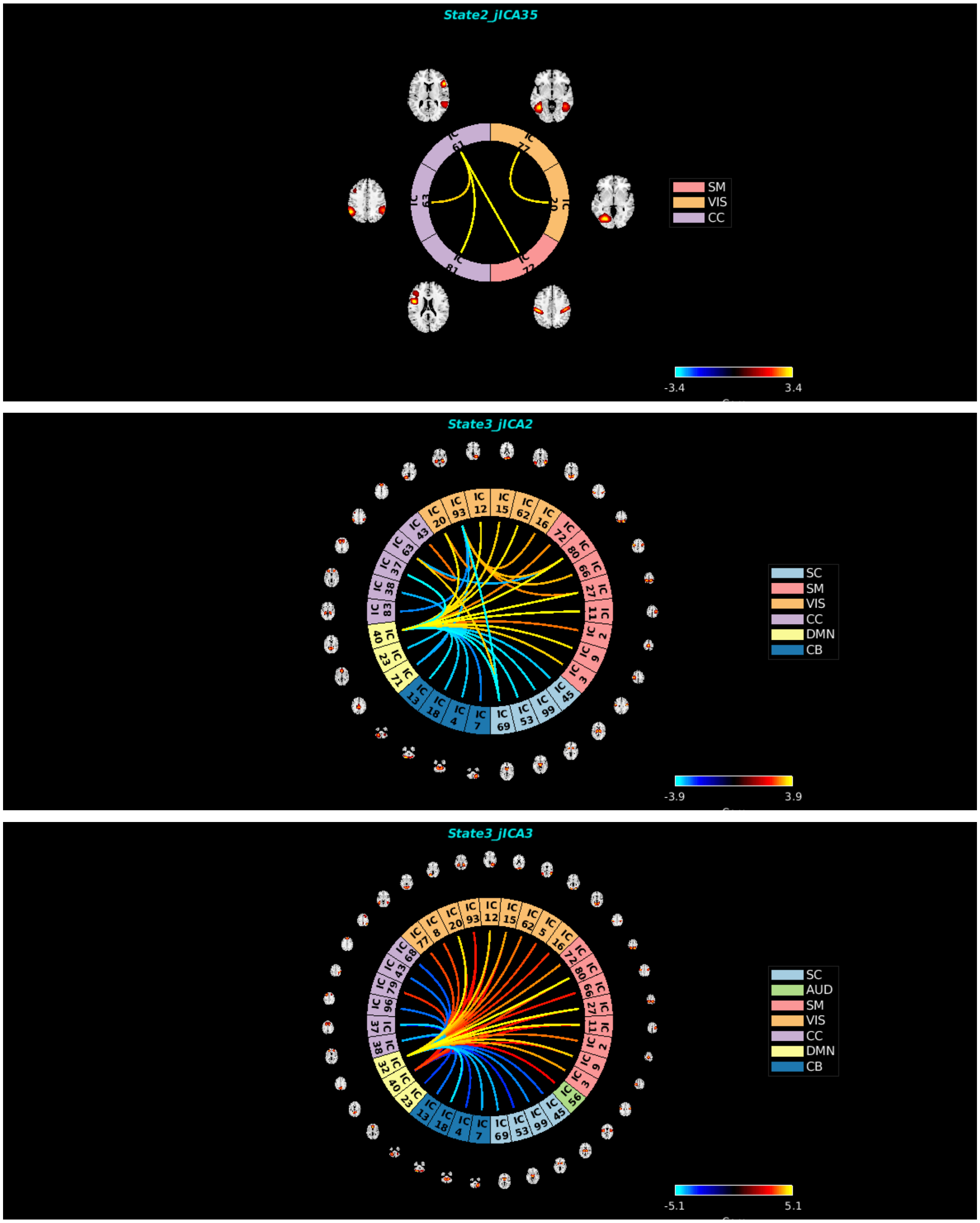
State2_jICA35
SM
VIS
CC
-3.4
3.4
State3_jICA2
SC
SM
VIS
CC
DMN
CB
-3.9
3.9
State3_jICA3
SC
AUD
SM
VIS
CC
DMN
CB
-5.1
5.1

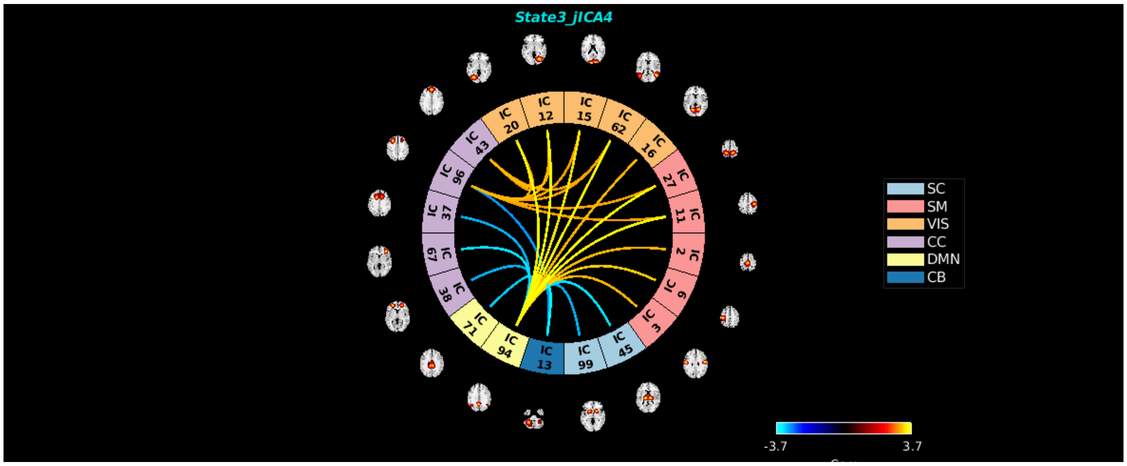
State3_jICA4
IC 20
IC 12
IC 15
IC 62
IC 16
IC 27
IC 11
IC 2
IC 9
IC 3
IC 45
IC 99
IC 13
IC 94
IC 71
IC 38
IC 67
IC 37
IC 96
IC 43
SC
SM
VIS
CC
DMN
CB
-3.7
3.7

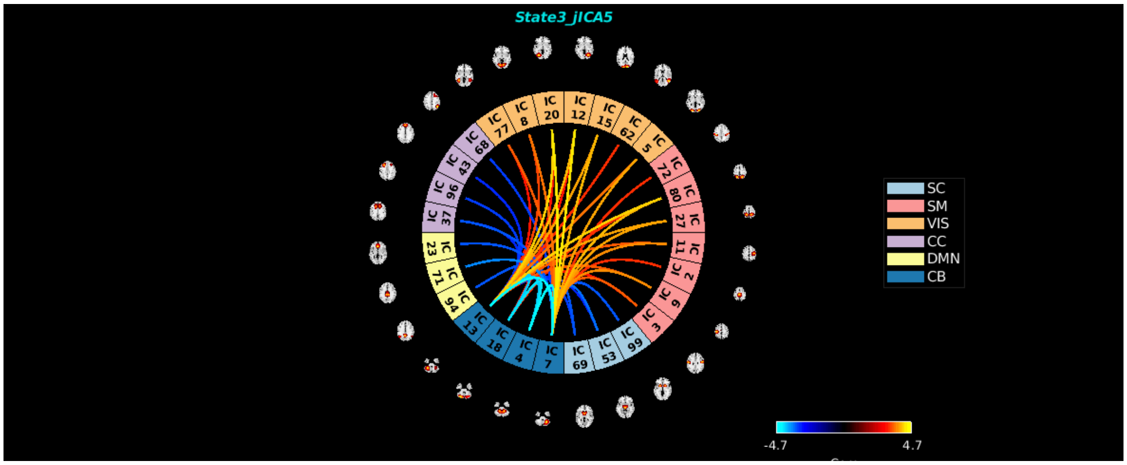
State3_jICA5
IC 77
IC 8
IC 20
IC 12
IC 15
IC 62
IC 5
IC 72
IC 80
IC 27
IC 11
IC 2
IC 9
IC 3
IC 99
IC 53
IC 69
IC 7
IC 4
IC 18
IC 13
IC 94
IC 71
IC 23
IC 37
IC 96
IC 43
IC 68
SC
SM
VIS
CC
DMN
CB
-4.7
4.7

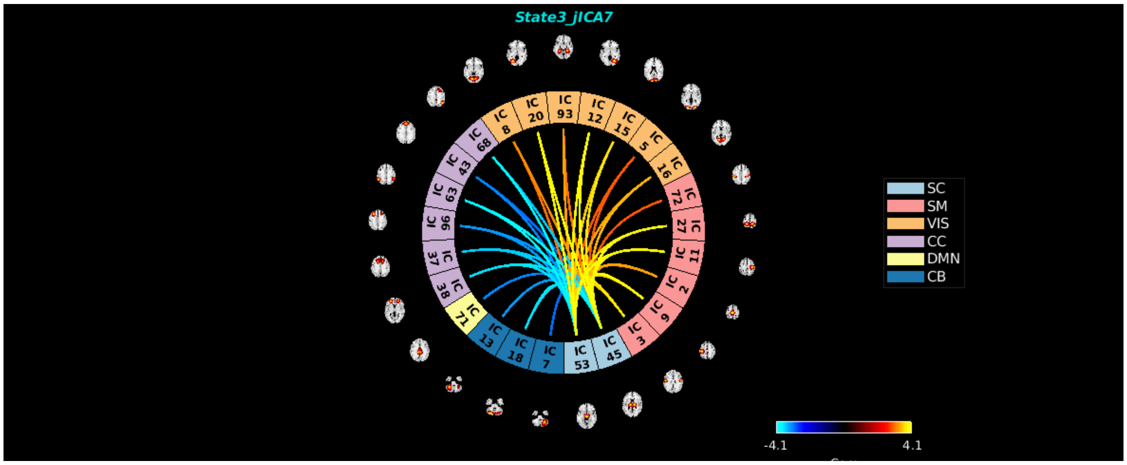
State3_jICA7
IC 8
IC 20
IC 93
IC 12
IC 15
IC 5
IC 16
IC 72
IC 27
IC 11
IC 2
IC 9
IC 3
IC 45
IC 53
IC 7
IC 18
IC 13
IC 71
IC 38
IC 37
IC 96
IC 63
IC 43
IC 68
SC
SM
VIS
CC
DMN
CB
-4.1
4.1

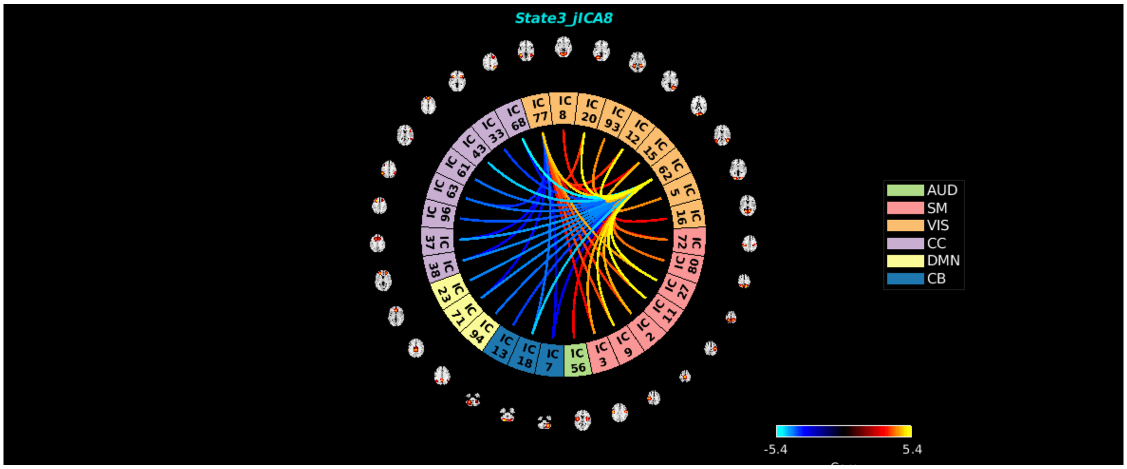
State3_jICA8
AUD
SM
VIS
CC
DMN
CB
-5.4
5.4

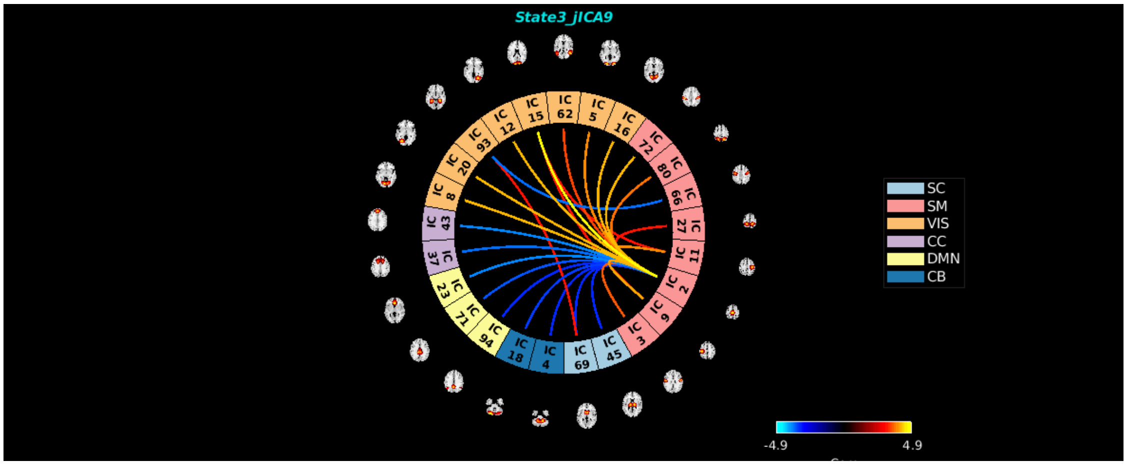
State3_jICA9
SC
SM
VIS
CC
DMN
CB
-4.9
4.9

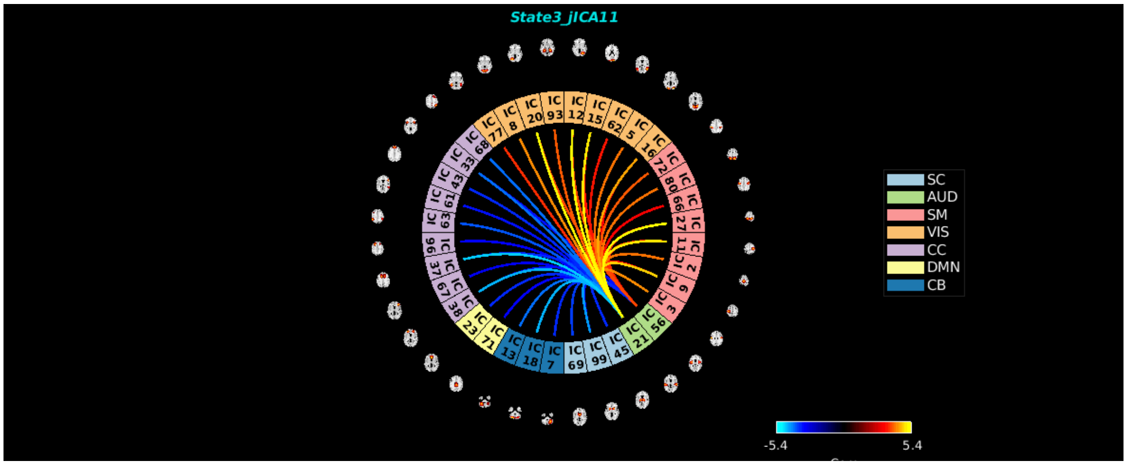
State3_jICA11
SC
AUD
SM
VIS
CC
DMN
CB
-5.4
5.4

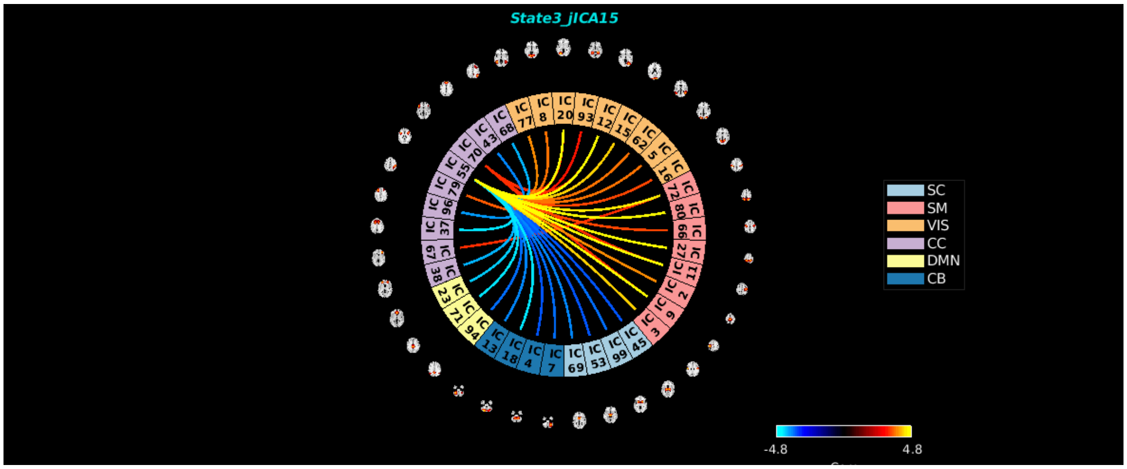
State3_jICA15
SC
SM
VIS
CC
DMN
CB
-4.8
4.8

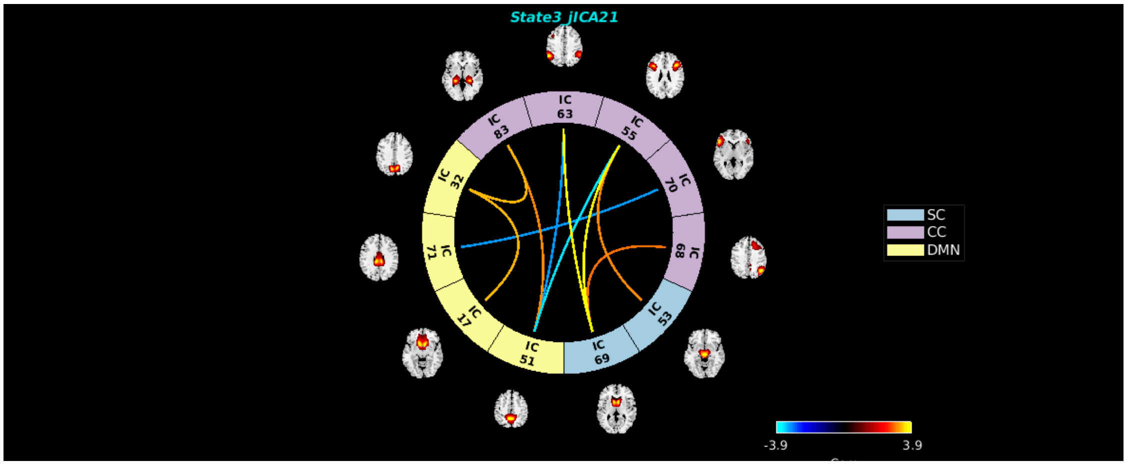
State3_jICA21
IC 63
IC 55
IC 70
IC 68
IC 53
IC 69
IC 51
IC 17
IC 71
IC 32
IC 83
SC
CC
DMN
-3.9
3.9

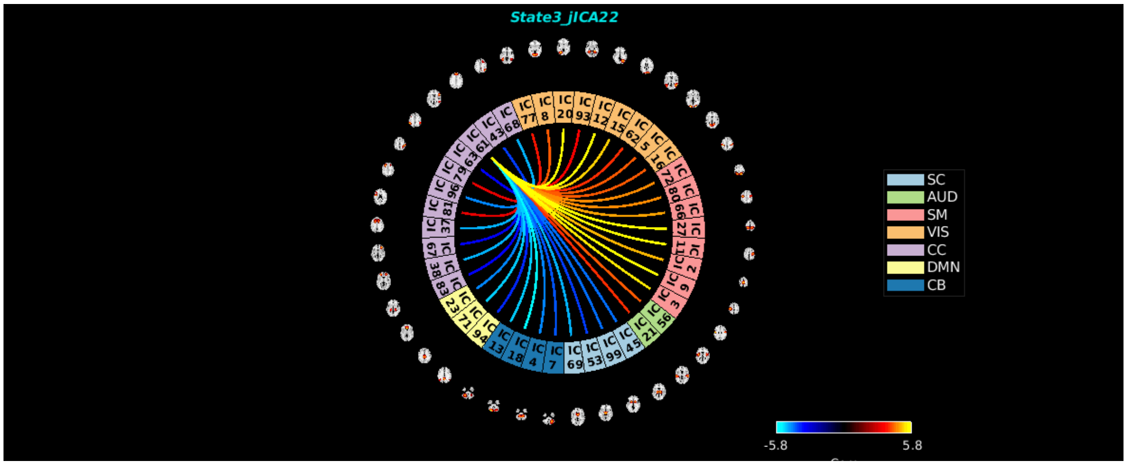
State3_jICA22
SC
AUD
SM
VIS
CC
DMN
CB
-5.8
5.8

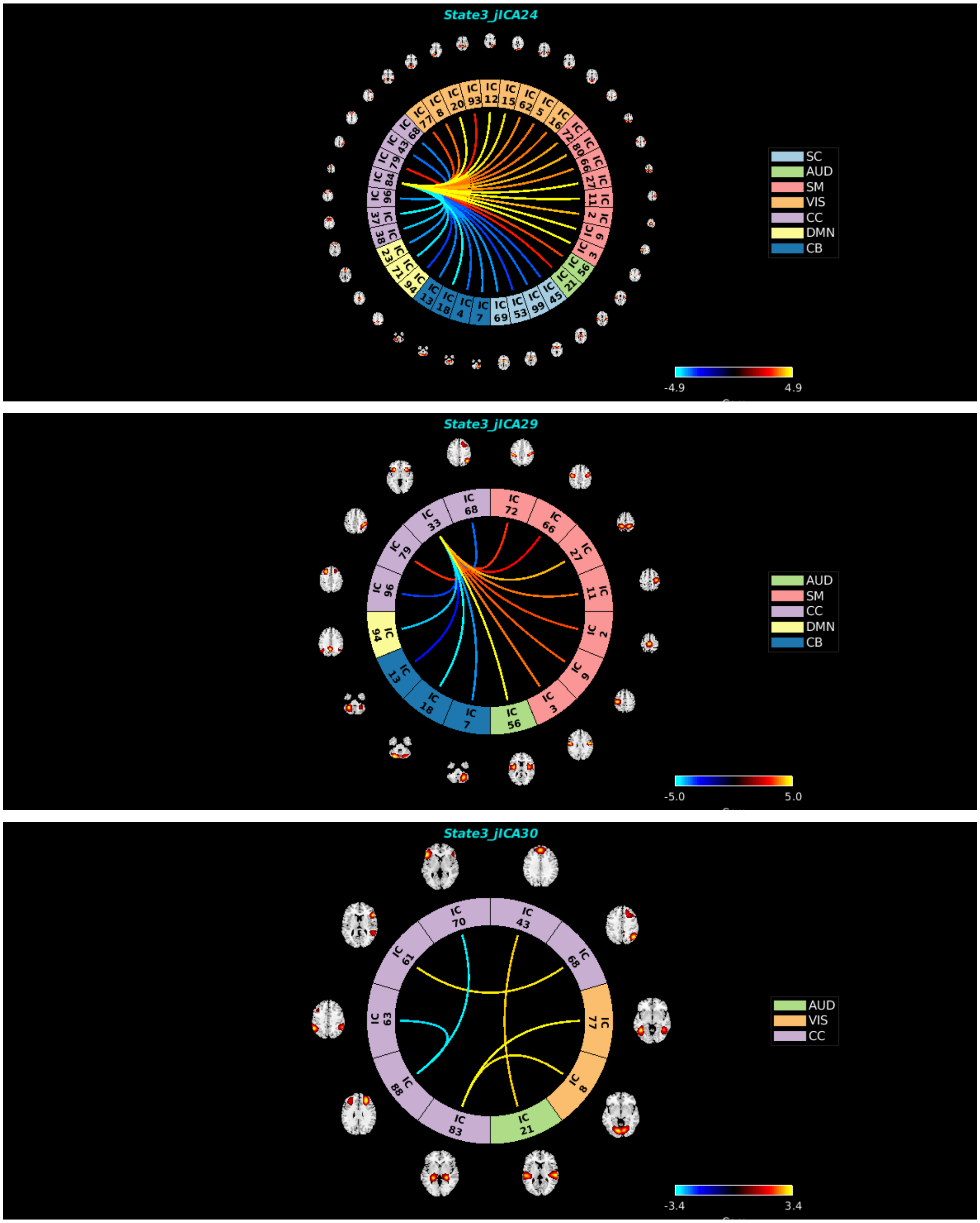
State3_jICA24
SC
AUD
SM
VIS
CC
DMN
CB
-4.9
4.9
State3_jICA29
AUD
SM
CC
DMN
CB
-5.0
5.0
State3_jICA30
AUD
VIS
CC
-3.4
3.4

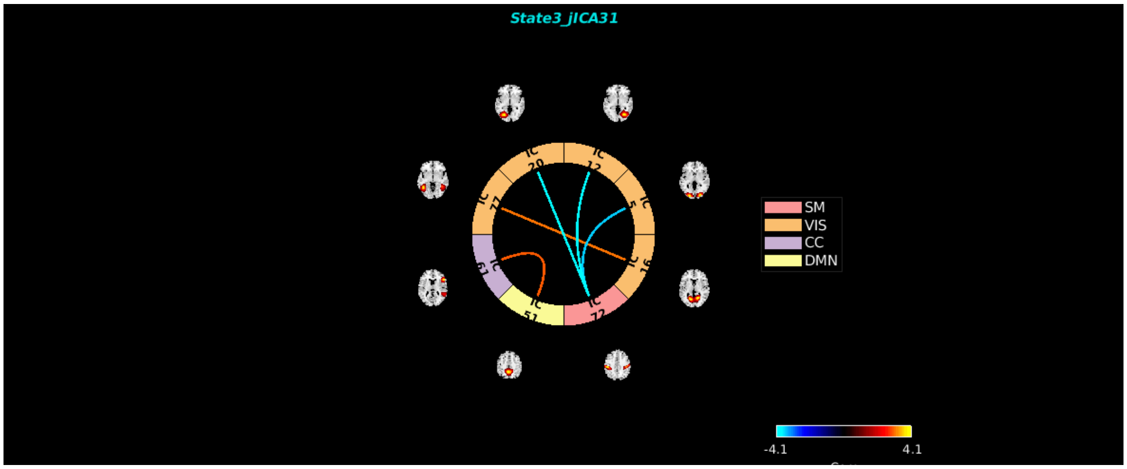
State3_jICA31
SM
VIS
CC
DMN
-4.1
4.1

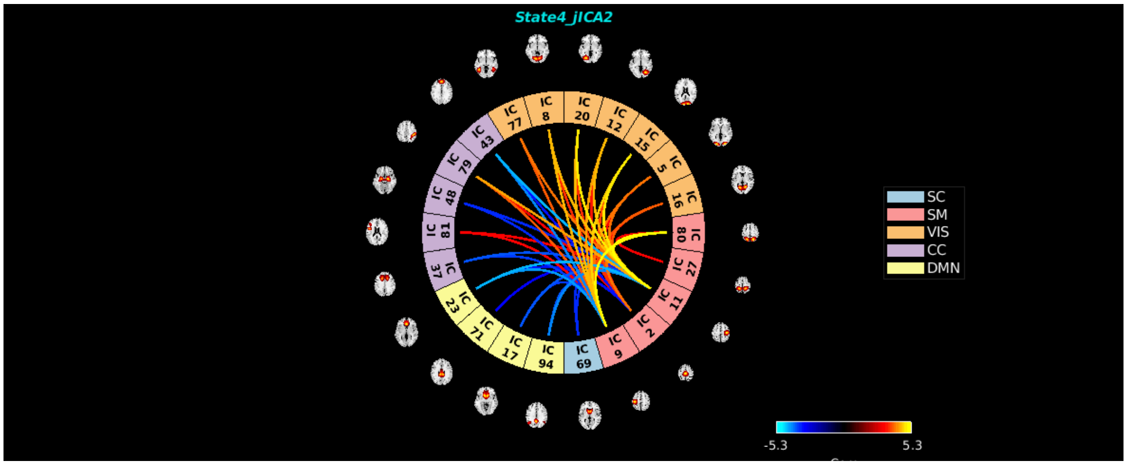
State4_jICA2
SC
SM
VIS
CC
DMN
-5.3
5.3

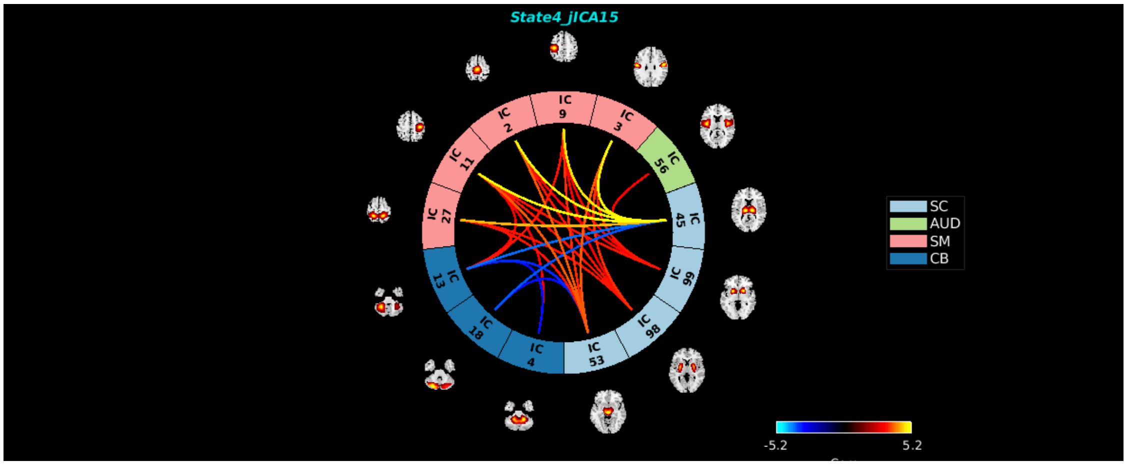
State4_jICA15
SC
AUD
SM
CB
-5.2
5.2

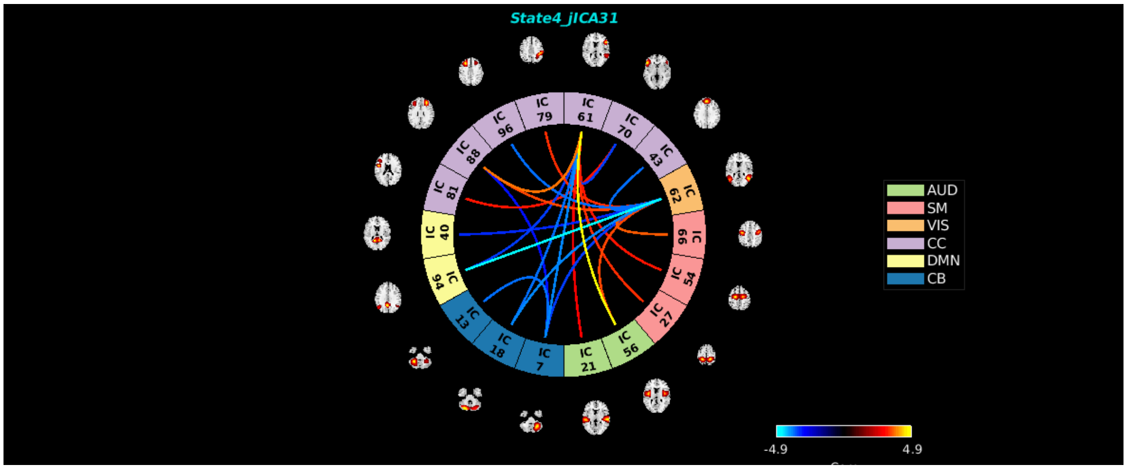
State4_jICA31
IC 79
IC 61
IC 96
IC 70
IC 88
IC 43
IC 81
IC 62
IC 40
IC 66
IC 94
IC 54
IC 13
IC 27
IC 18
IC 7
IC 21
IC 56
AUD
SM
VIS
CC
DMN
CB
-4.9
4.9
