# Supplementary material for: Dynamic Fusion of Genomics and Functional Network Connectivity in UK Biobank Reveals Schizophrenia‐Related SNP Manifolds
Source: Hum Brain Mapp. 2026 Apr 20;47(6):e70530. doi: 10.1002/hbm.70530 (PMC13095861; doi:10.1002/hbm.70530)
Supplement: Supplementary file 2 — Figure S2: Manhattan plot of the top SNPs for each of the identified schizophrenia relevant components. [file HBM-47-e70530-s002.pdf]

Figure S2: Manhattan plot of the top SNPs for each of the identified schizophrenia-relevant components.

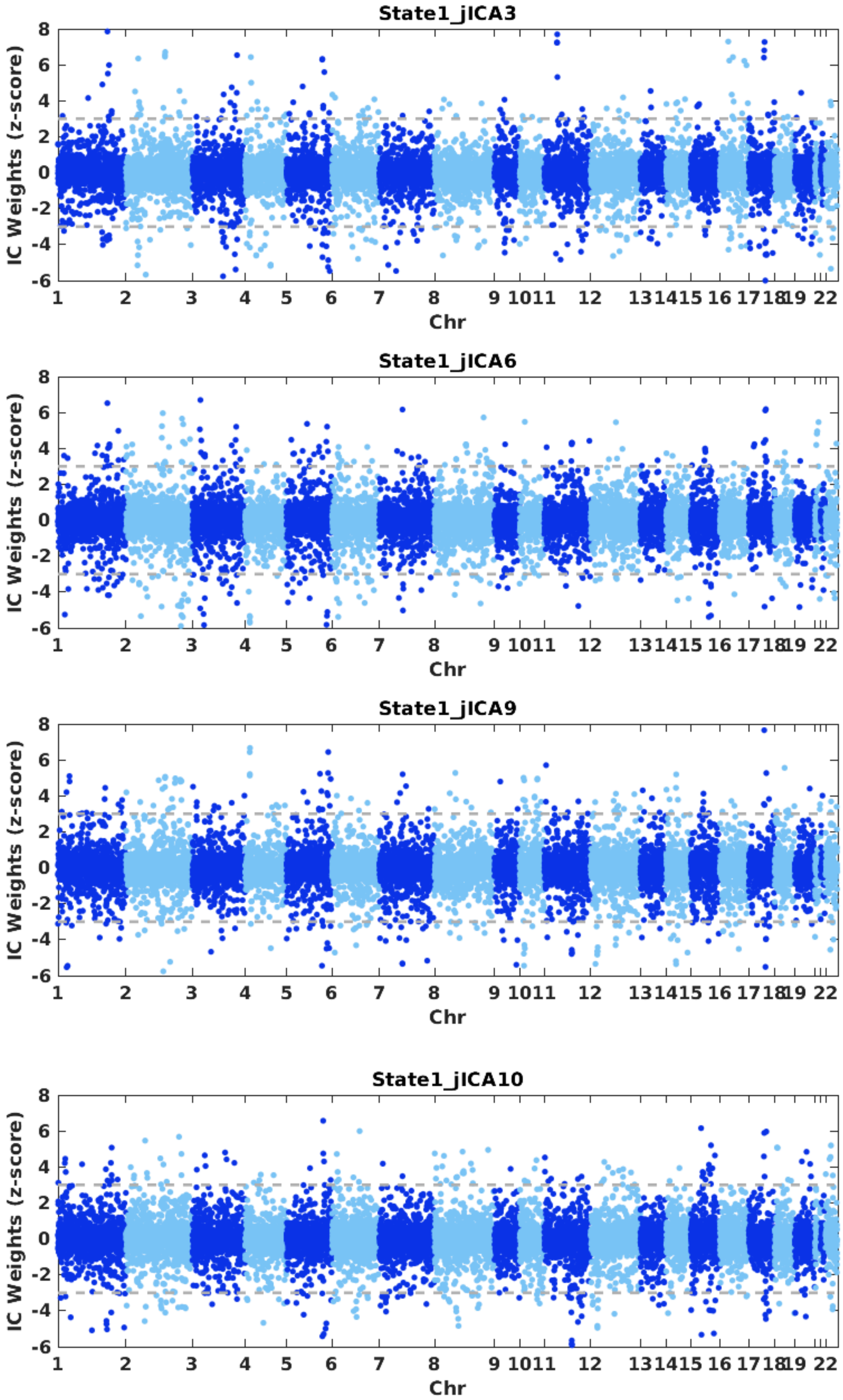

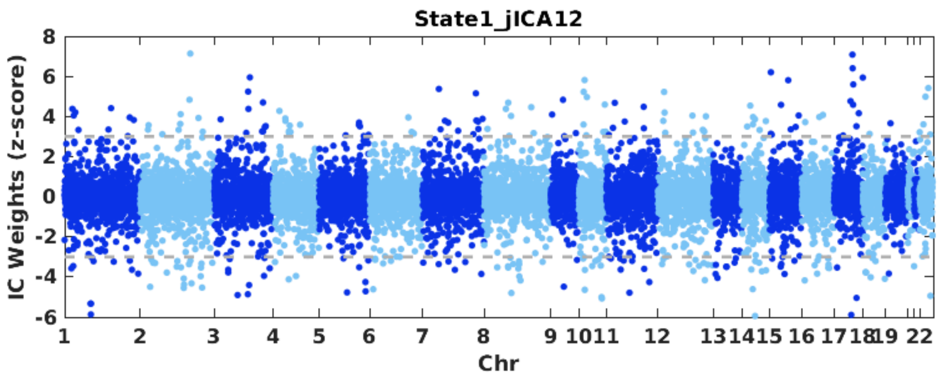
State1_jICA12
IC Weights (z-score)
Chr
1 2 3 4 5 6 7 8 9 1011 12 131415 16 171819 22

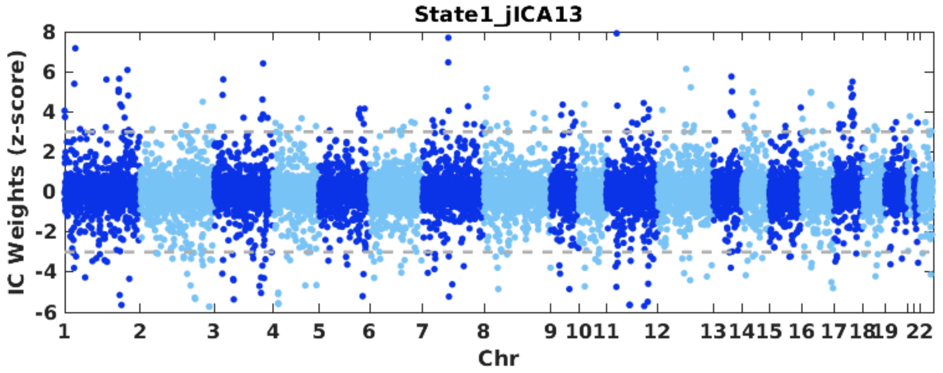
State1_jICA13
IC Weights (z-score)
Chr
1 2 3 4 5 6 7 8 9 1011 12 131415 16 171819 22

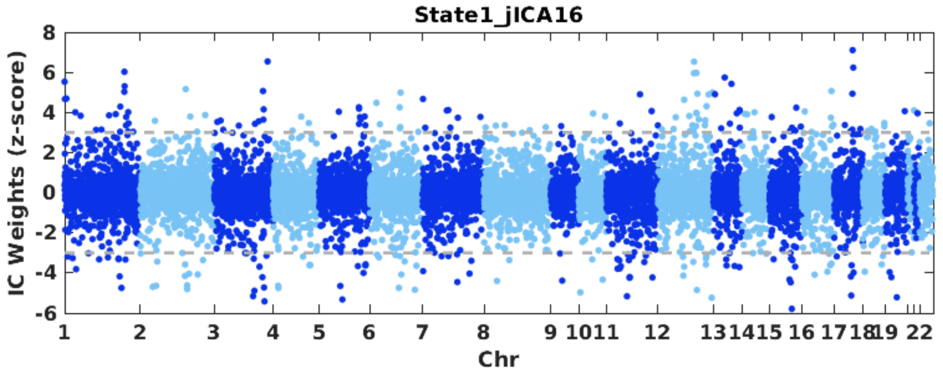
State1_jICA16
IC Weights (z-score)
Chr
1 2 3 4 5 6 7 8 9 1011 12 131415 16 171819 22

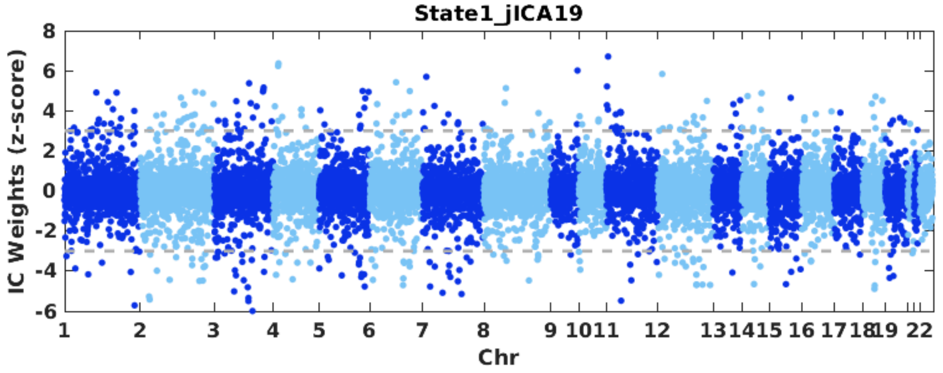
State1_jICA19
IC Weights (z-score)
Chr
1 2 3 4 5 6 7 8 9 1011 12 131415 16 171819 22

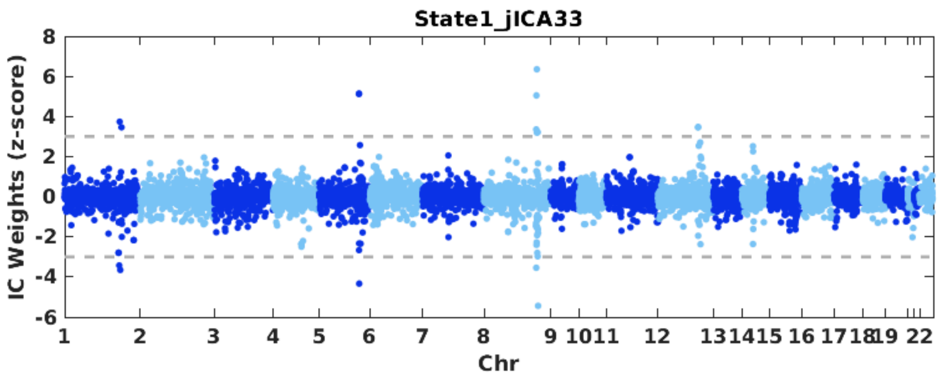
State1_jICA33
IC Weights (z-score)
Chr
1 2 3 4 5 6 7 8 9 10 11 12 13 14 15 16 17 18 19 22

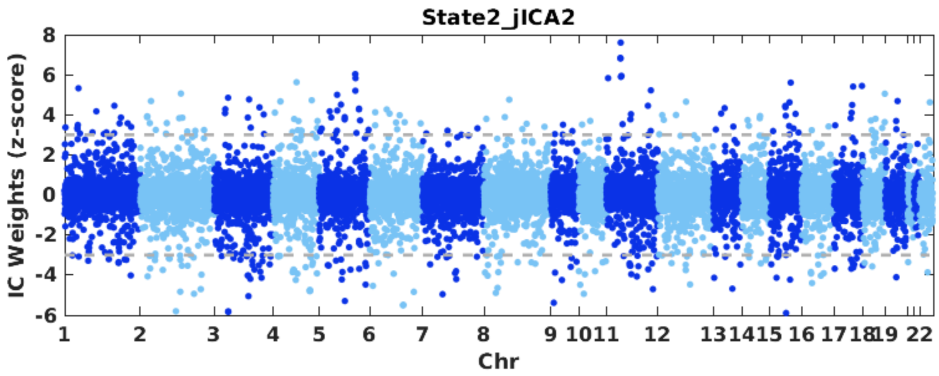
State2_jICA2
IC Weights (z-score)
Chr
1 2 3 4 5 6 7 8 9 10 11 12 13 14 15 16 17 18 19 22

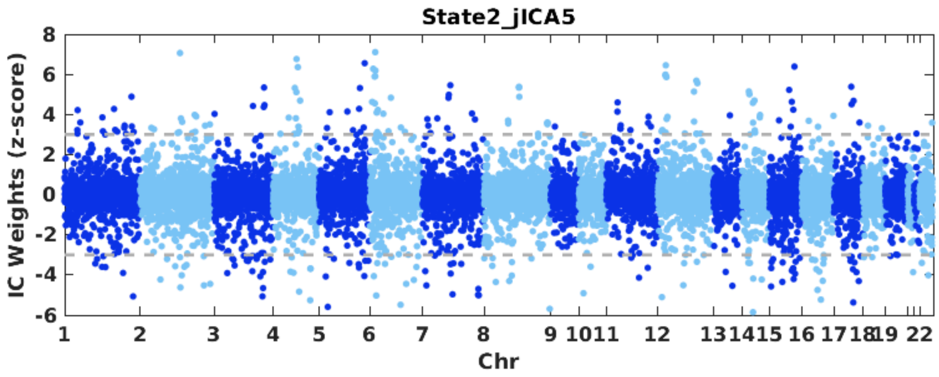
State2_jICA5
IC Weights (z-score)
Chr
1 2 3 4 5 6 7 8 9 10 11 12 13 14 15 16 17 18 19 22

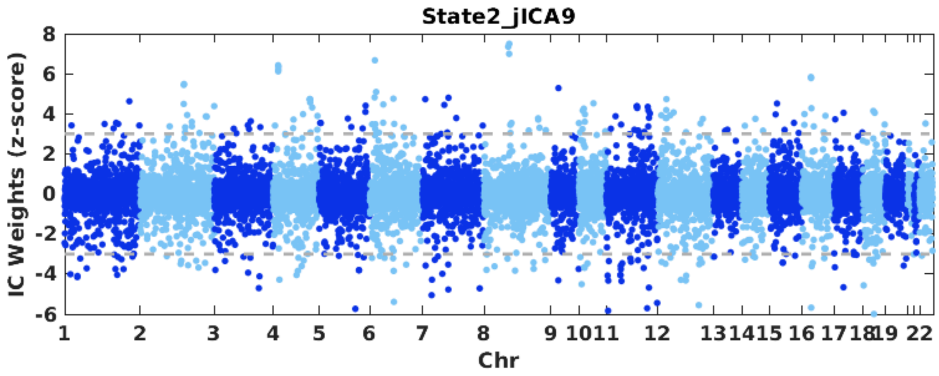
State2_jICA9
IC Weights (z-score)
Chr
1 2 3 4 5 6 7 8 9 10 11 12 13 14 15 16 17 18 19 22

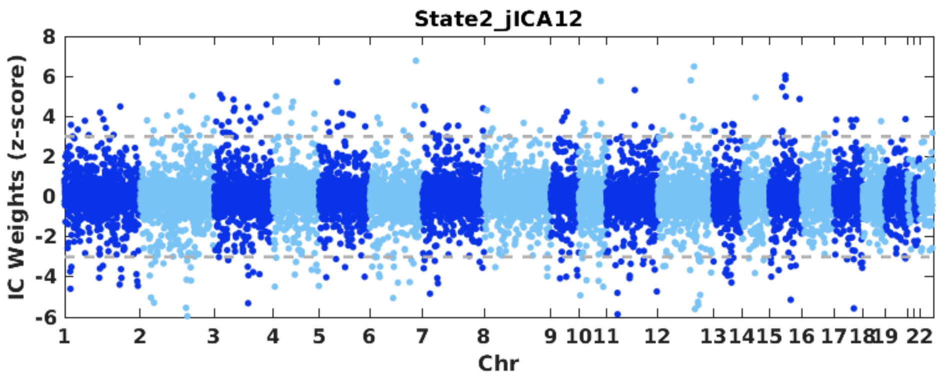
State2_jICA12
IC Weights (z-score)
Chr

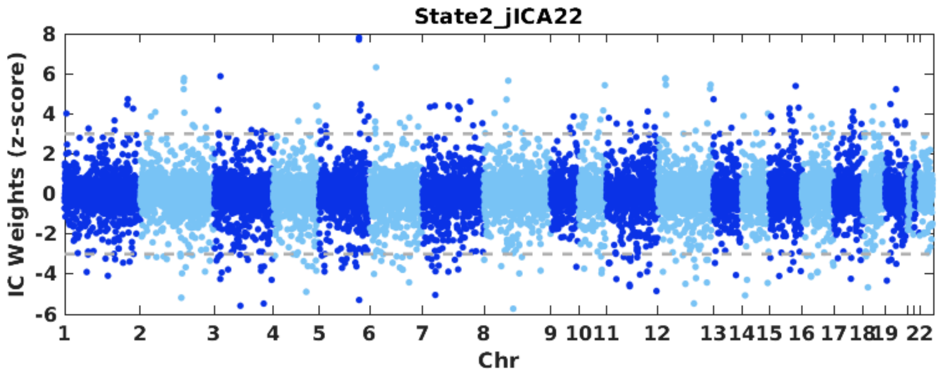
State2_jICA22
IC Weights (z-score)
Chr

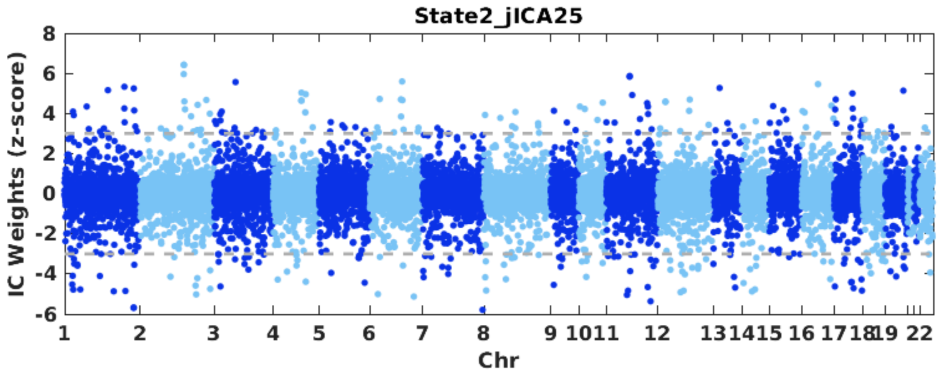
State2_jICA25
IC Weights (z-score)
Chr

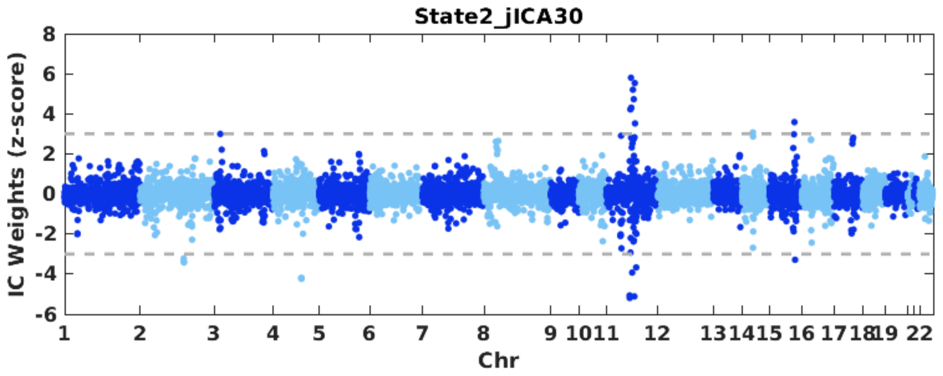
State2_jICA30
IC Weights (z-score)
Chr

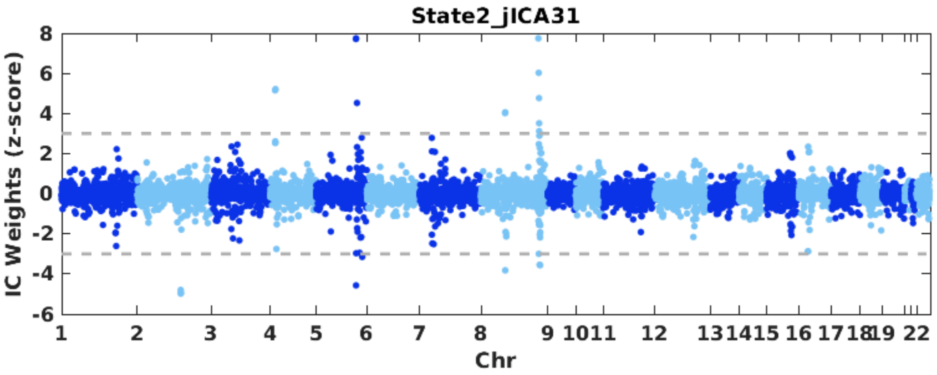
State2_jICA31
IC Weights (z-score)
8 6 4 2 0 -2 -4 -6
1 2 3 4 5 6 7 8 9 1011 12 131415 16 171819 22
Chr

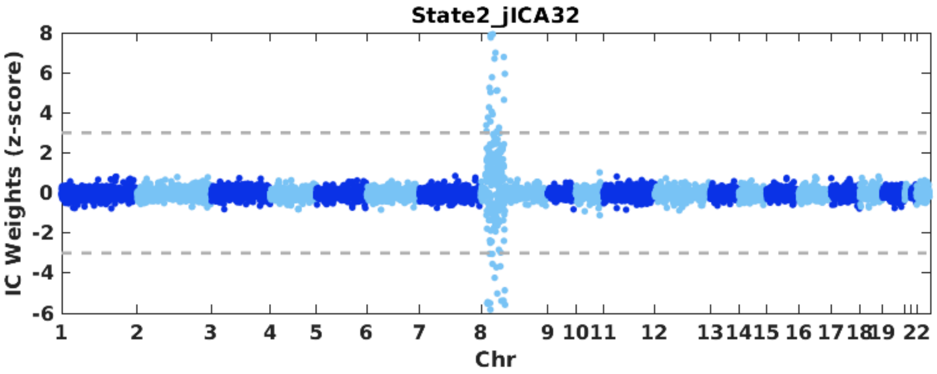
State2_jICA32
IC Weights (z-score)
8 6 4 2 0 -2 -4 -6
1 2 3 4 5 6 7 8 9 1011 12 131415 16 171819 22
Chr

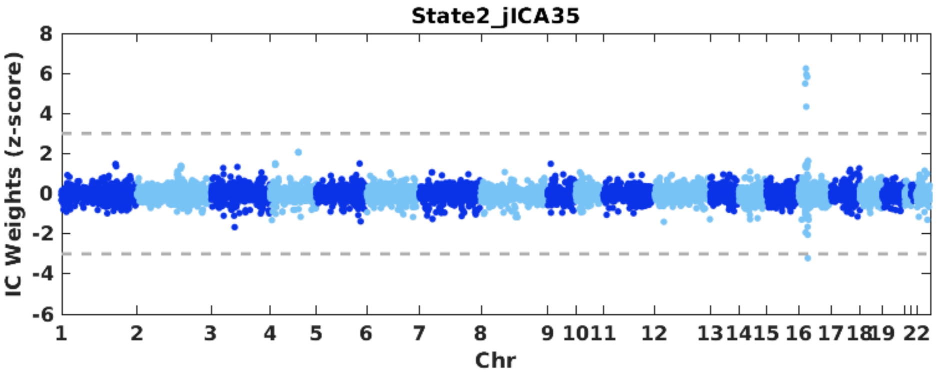
State2_jICA35
IC Weights (z-score)
8 6 4 2 0 -2 -4 -6
1 2 3 4 5 6 7 8 9 1011 12 131415 16 171819 22
Chr

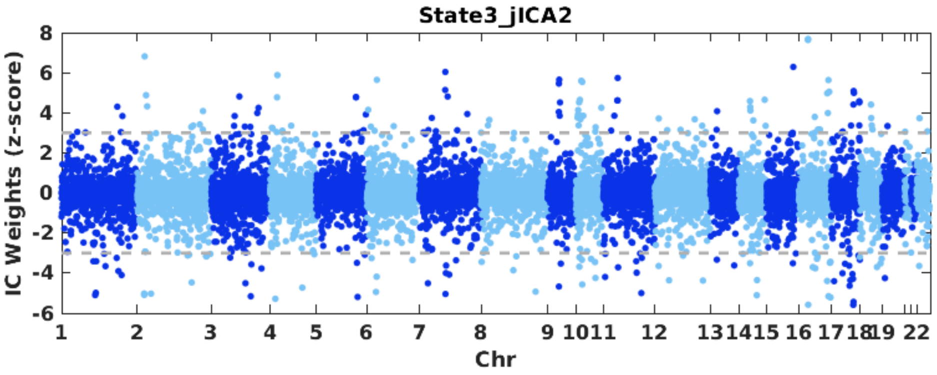
State3_jICA2
IC Weights (z-score)
8 6 4 2 0 -2 -4 -6
1 2 3 4 5 6 7 8 9 1011 12 131415 16 171819 22
Chr

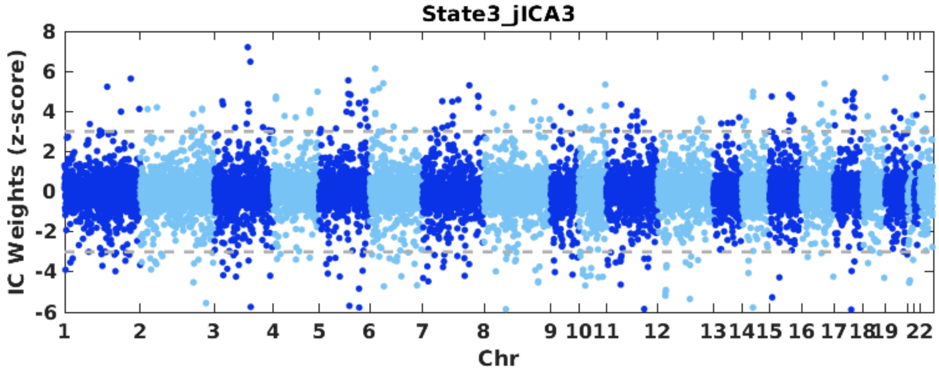
State3_jICA3
IC Weights (z-score)
Chr
1 2 3 4 5 6 7 8 9 1011 12 131415 16 171819 22

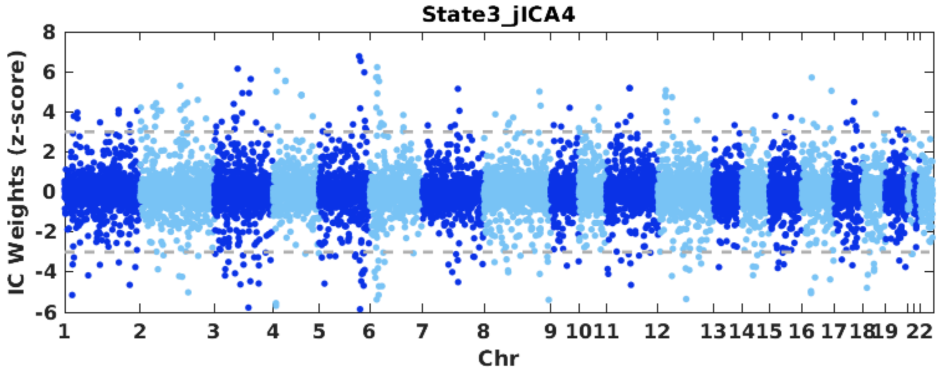
State3_jICA4
IC Weights (z-score)
Chr
1 2 3 4 5 6 7 8 9 1011 12 131415 16 171819 22

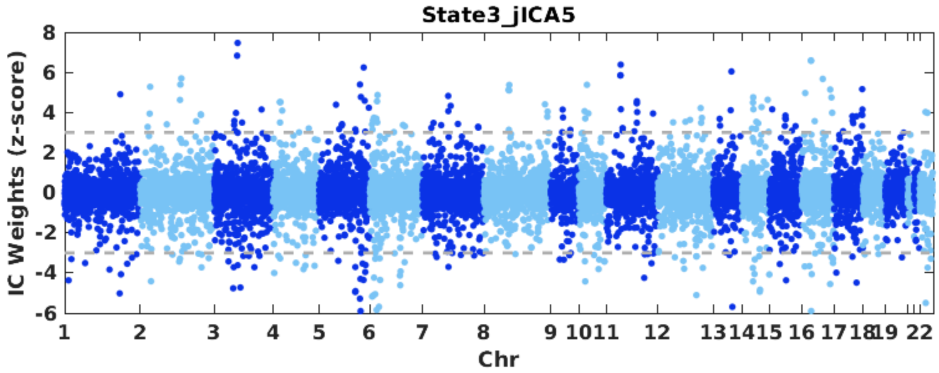
State3_jICA5
IC Weights (z-score)
Chr
1 2 3 4 5 6 7 8 9 1011 12 131415 16 171819 22

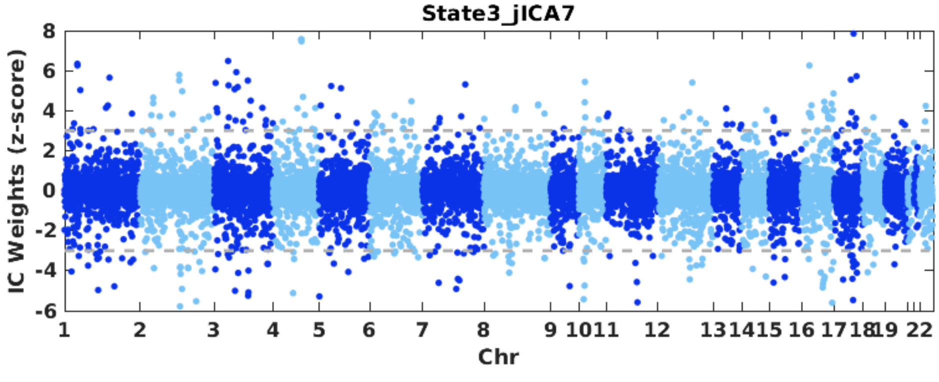
State3_jICA7
IC Weights (z-score)
Chr
1 2 3 4 5 6 7 8 9 1011 12 131415 16 171819 22

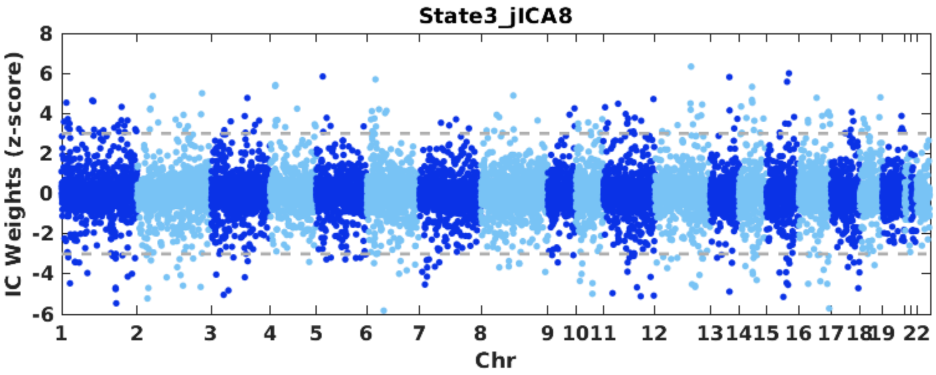
State3_jICA8
IC Weights (z-score)
8 6 4 2 0 -2 -4 -6
1 2 3 4 5 6 7 8 9 1011 12 131415 16 171819 22
Chr

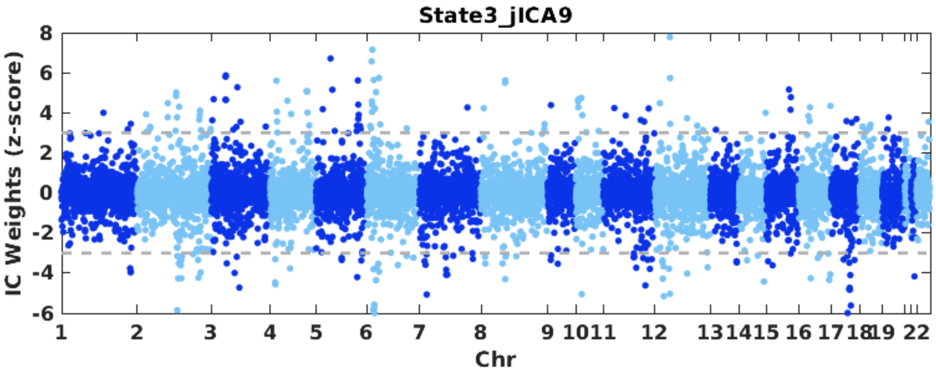
State3_jICA9
IC Weights (z-score)
8 6 4 2 0 -2 -4 -6
1 2 3 4 5 6 7 8 9 1011 12 131415 16 171819 22
Chr

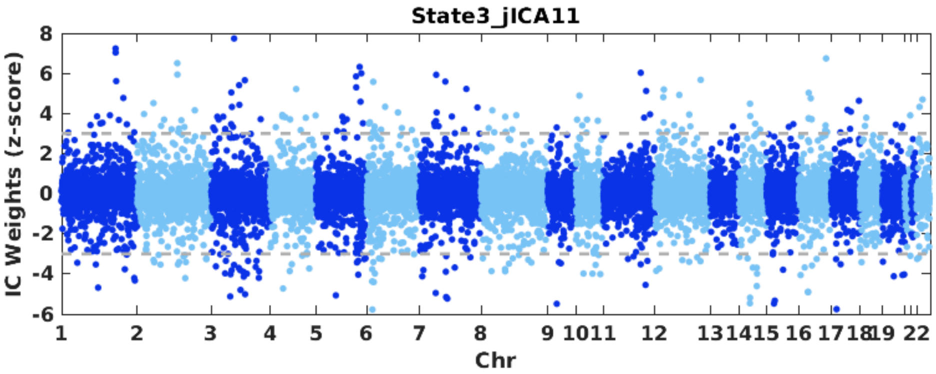
State3_jICA11
IC Weights (z-score)
8 6 4 2 0 -2 -4 -6
1 2 3 4 5 6 7 8 9 1011 12 131415 16 171819 22
Chr

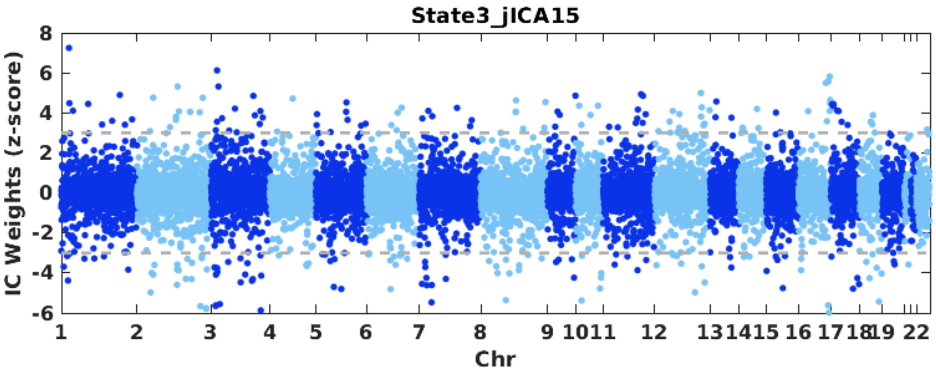
State3_jICA15
IC Weights (z-score)
8 6 4 2 0 -2 -4 -6
1 2 3 4 5 6 7 8 9 1011 12 131415 16 171819 22
Chr

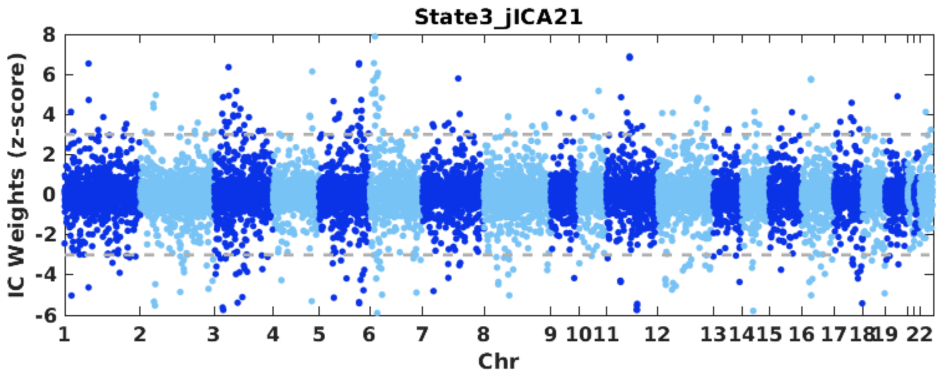

State3_jICA21
IC Weights (z-score)
Chr

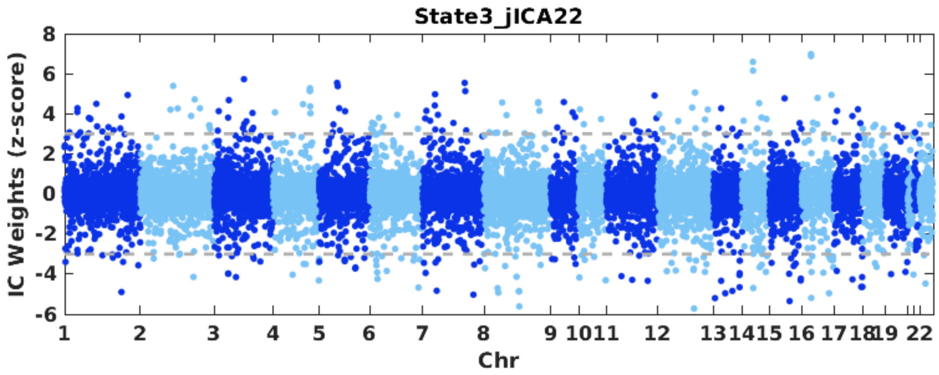

State3_jICA22
IC Weights (z-score)
Chr

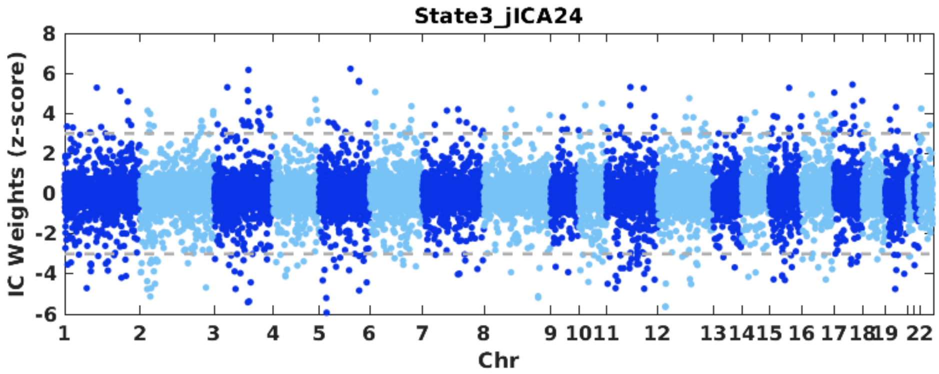

State3_jICA24
IC Weights (z-score)
Chr

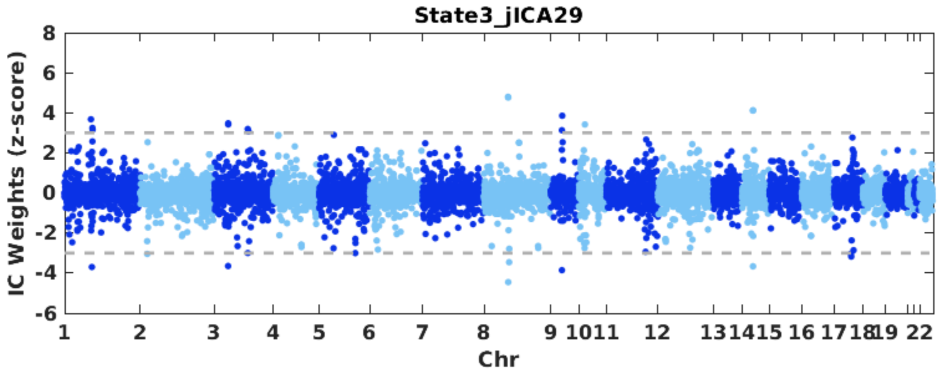

State3_jICA29
IC Weights (z-score)
Chr

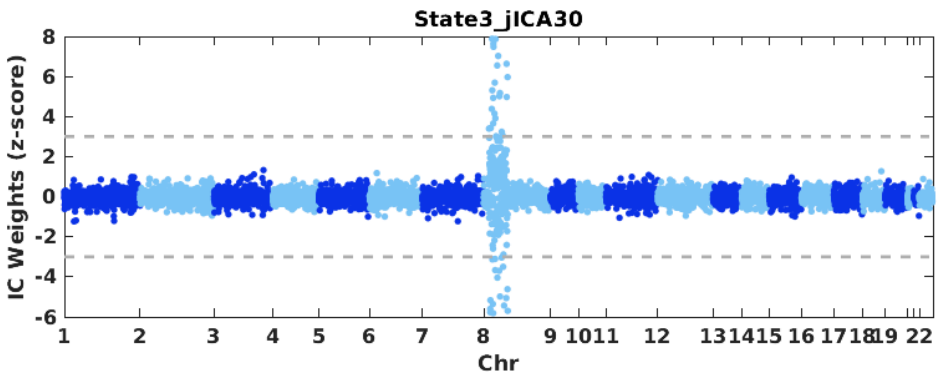
State3_jICA30
IC Weights (z-score)
Chr

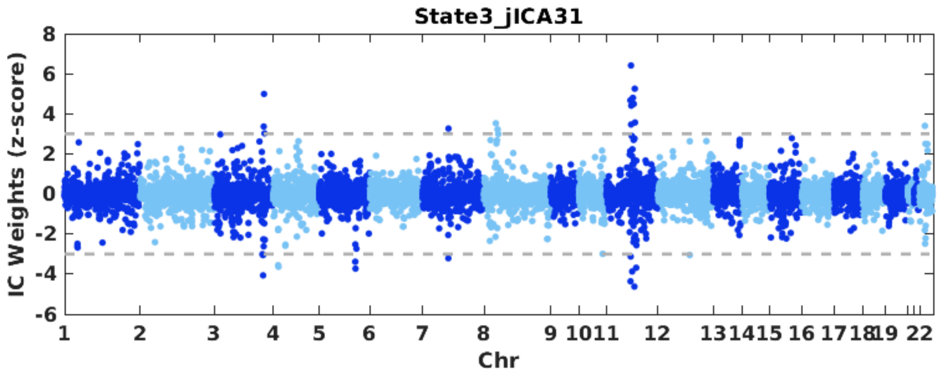
State3_jICA31
IC Weights (z-score)
Chr

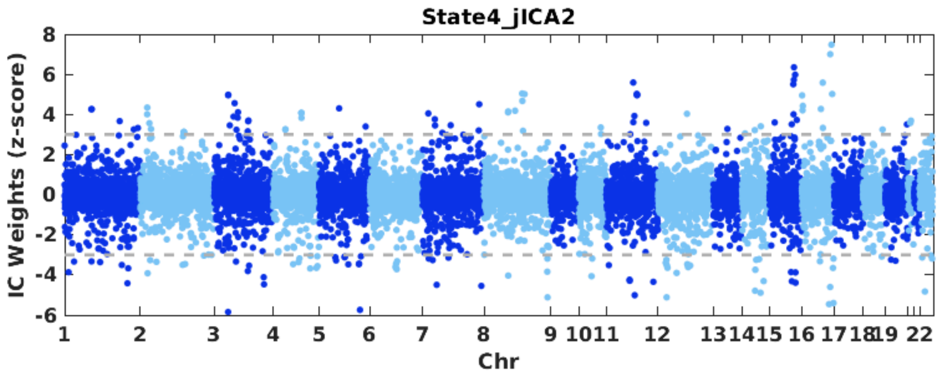
State4_jICA2
IC Weights (z-score)
Chr

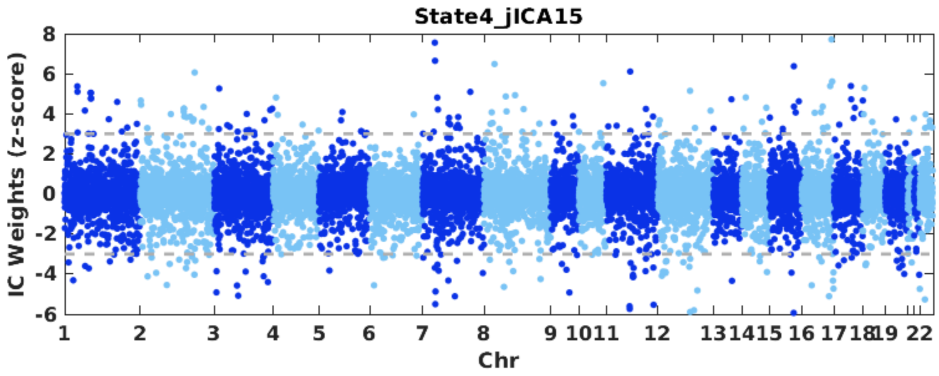
State4_jICA15
IC Weights (z-score)
Chr

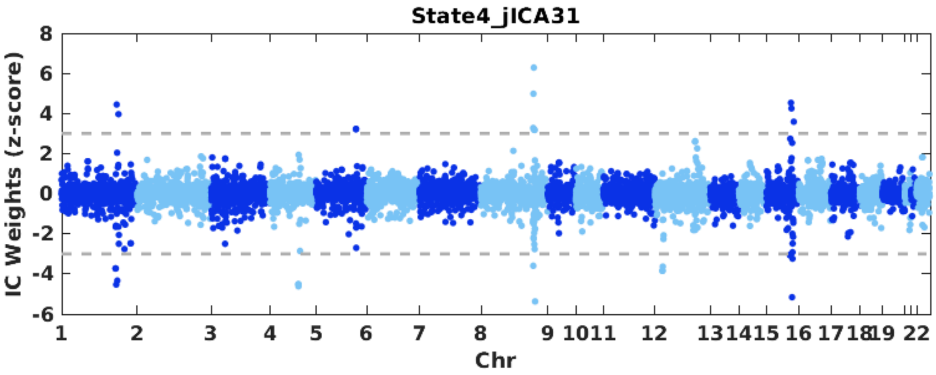
State4_jICA31
IC Weights (z-score)
8
6
4
2
0
-2
-4
-6
1
2
3
4
5
6
7
8
9
10
11
12
13
14
15
16
17
18
19
22
Chr
